# A Bacteriophage-Encoded J-Domain Protein Interacts with the DnaK/Hsp70 Chaperone and Stabilizes the Heat-Shock Factor σ^32^ of *Escherichia coli*

**DOI:** 10.1371/journal.pgen.1003037

**Published:** 2012-11-01

**Authors:** Elsa Perrody, Anne-Marie Cirinesi, Carine Desplats, France Keppel, Françoise Schwager, Samuel Tranier, Costa Georgopoulos, Pierre Genevaux

**Affiliations:** 1 Laboratoire de Microbiologie et Génétique Moléculaire (LMGM), UMR5100, Centre National de la Recherche Scientifique (CNRS) and Université Paul Sabatier, Toulouse, France; 2 Département de Microbiologie et Médecine Moléculaire, CMU, Université de Genève, Geneva, Switzerland; 3 Institut de Pharmacologie et de Biologie Structurale, Centre National de la Recherche Scientifique (CNRS), Toulouse, France; 4 Department of Biochemistry, University of Utah School of Medicine, Salt Lake City, Utah, United States of America; Universidad de Sevilla, Spain

## Abstract

The universally conserved J-domain proteins (JDPs) are obligate cochaperone partners of the Hsp70 (DnaK) chaperone. They stimulate Hsp70's ATPase activity, facilitate substrate delivery, and confer specific cellular localization to Hsp70. In this work, we have identified and characterized the first functional JDP protein encoded by a bacteriophage. Specifically, we show that the ORFan gene *057w* of the T4-related enterobacteriophage RB43 encodes a *bona fide* JDP protein, named Rki, which specifically interacts with the *Escherichia coli* host multifunctional DnaK chaperone. However, in sharp contrast with the three known host JDP cochaperones of DnaK encoded by *E. coli*, Rki does not act as a generic cochaperone *in vivo* or *in vitro*. Expression of Rki alone is highly toxic for wild-type *E. coli*, but toxicity is abolished in the absence of endogenous DnaK or when the conserved J-domain of Rki is mutated. Further *in vivo* analyses revealed that Rki is expressed early after infection by RB43 and that deletion of the *rki* gene significantly impairs RB43 proliferation. Furthermore, we show that mutations in the host *dnaK* gene efficiently suppress the growth phenotype of the RB43 *rki* deletion mutant, thus indicating that Rki specifically interferes with DnaK cellular function. Finally, we show that the interaction of Rki with the host DnaK chaperone rapidly results in the stabilization of the heat-shock factor σ^32^, which is normally targeted for degradation by DnaK. The mechanism by which the Rki-dependent stabilization of σ^32^ facilitates RB43 bacteriophage proliferation is discussed.

## Author Summary

Bacteriophages are the most abundant biological entities on earth. As a consequence, they represent the largest reservoir of unexplored genetic information. They control bacterial growth, mediate horizontal gene transfer, and thus exert profound influence on microbial ecology and growth. One of the striking features of bacteriophages is that they code for many open reading frames of thus far unknown biological function (called ORFans), which have been referred to as the dark matter of our biosphere. Here we have extensively characterized such a novel ORFan-encoded protein, Rki, encoded by the large, virulent enterobacteriaceae bacteriophage RB43. We show that Rki functions to control the host stress-response during the early stages of bacteriophage infection, specifically by interacting with the host DnaK/Hsp70 chaperone to stabilize the major host heat-shock factor, σ^32^.

## Introduction

The universally conserved molecular chaperone machines maintain cellular protein homeostasis by acting at almost every stage in the life of proteins [1]. In the bacterium *Escherichia coli*, the multifunctional DnaK (Hsp70) chaperone machine (the DnaK/DnaJ/GrpE complex) performs key cellular functions under both physiological and stress conditions [2]–[4]. For example, it assists *de novo* protein folding and targeting to biological membranes, protein quality control, assembly or disassembly of oligomeric complexes, and signal transduction. It responds to multiple stresses leading to protein misfolding and aggregation [5]. Moreover, the DnaK machine controls the entire *E. coli* heat-shock response by binding specifically to the major stress sigma factor σ^32^ and facilitating its degradation by the membrane-anchored FtsH protease [6]. The multiple phenotypes associated with the loss of DnaK in *E. coli* attest to its central role in protein biogenesis [7].

The DnaK protein is composed of an N-terminal nucleotide-binding domain and a C-terminal substrate-binding domain connected by a conserved linker involved in conformational changes and stability [8]–[10]. While the ATP-bound DnaK exhibits a low affinity and fast exchange rate for its substrate, the ADP-bound form is characterized by high affinity and low exchange rates. Specific cochaperones regulate its switch from one state to the other, thus coordinating DnaK's various intracellular functions. For example, the cochaperone DnaJ (Hsp40) stimulates DnaK's ATPase activity and targets specific substrates to DnaK [11], resulting in the formation of a stable ADP-bound DnaK-substrate complex [12]. The nucleotide exchange factor GrpE stimulates ADP-ATP exchange by triggering substrate release, thus resetting DnaK's cycle [13].

All the DnaJ/Hsp40 cochaperones are characterized by the presence of a compact domain of about 70 amino acids, called the J-domain, which is essential for a functional interaction with Hsp70. Therefore, these cochaperones are generally called JDPs for J-domain proteins [14]. JDPs have been divided into three classes. Adjacent to their J-domain, type I JDPs share a G/F-rich region, a zinc-binding domain and a C-terminal domain involved in substrate-binding [15]. Type II JDPs generally have a similar domain arrangement except that they do not possess a zinc-binding domain [16]. The type I and type II JDPs generally bind a large variety of unfolded substrates in response to stress and are thus considered generic cochaperones [17]. In contrast, type III JDP members only have the J-domain in common with the other JDPs, suggesting that they deliver specific substrates or confer specific cellular localization to Hsp70 [18]. In *E. coli*, the chaperone DnaK interacts with the type I and II JDPs, DnaJ and CbpA, respectively, as well as with the type III protein DjlA, which may confer to DnaK its membrane localization [7], [19].

In general, the successful proliferation of viruses relies on both the efficient reprogramming of the host cell cycle and the rapid synthesis and subsequent folding of a large number of viral proteins necessary for genome replication, protein synthesis and capsid assembly [20]. Since molecular chaperones are generally involved in both processes, it is not surprising that many viruses utilize the host cellular chaperones at different stages in their life cycle. Among these chaperones, Hsp70 (DnaK) is often recruited by eukaryotic viruses to assist viral entry, replication, gene expression, folding and assembly of viral proteins, and to control the host cell cycle progression [21], [22]. Some viruses, such as polyoma and Molluscum contagiosum, encode their own JDPs to hijack the host Hsp70 chaperone [22]. To date, the best characterized virus-encoded JDPs are the type III viral T-antigens from simian virus 40 (SV40), which use their N-terminal J-domain for transcriptional activation of viral genes, viral DNA replication and capsid morphogenesis, as well as modulation of the host growth control signaling pathways to facilitate viral replication (review by [23]).

Putative gene products showing sequence similarities with the J-domain also can be found in some mycobacteriophage and enterobacteriophage genomes ([24], [25]; http://phage.ggc.edu). In this work, we have identified and characterized the first functional bacteriophage-encoded JDP protein. We show that the ORFan gene *057w* from the T4-related enterobacteriophage RB43, encodes a *bona fide* type III DnaJ-like protein, named Rki, which specifically interacts with the *E. coli* host multifunctional DnaK chaperone. However, in contrast with other JDPs, Rki expression is highly toxic for *E. coli* growth and this toxicity is fully dependent on the presence of endogenous DnaK. Analysis of the *rki* mutant bacteriophage further revealed that interaction with DnaK is critical for RB43 proliferation. Finally, we show that recruitment of the host DnaK chaperone by Rki rapidly results in the stabilization of the heat-shock factor σ^32^, thus facilitating bacteriophage proliferation.

## Results/Discussion

### The putative *057w* gene product of RB43 possesses a functional J-domain

**Figure 1:**
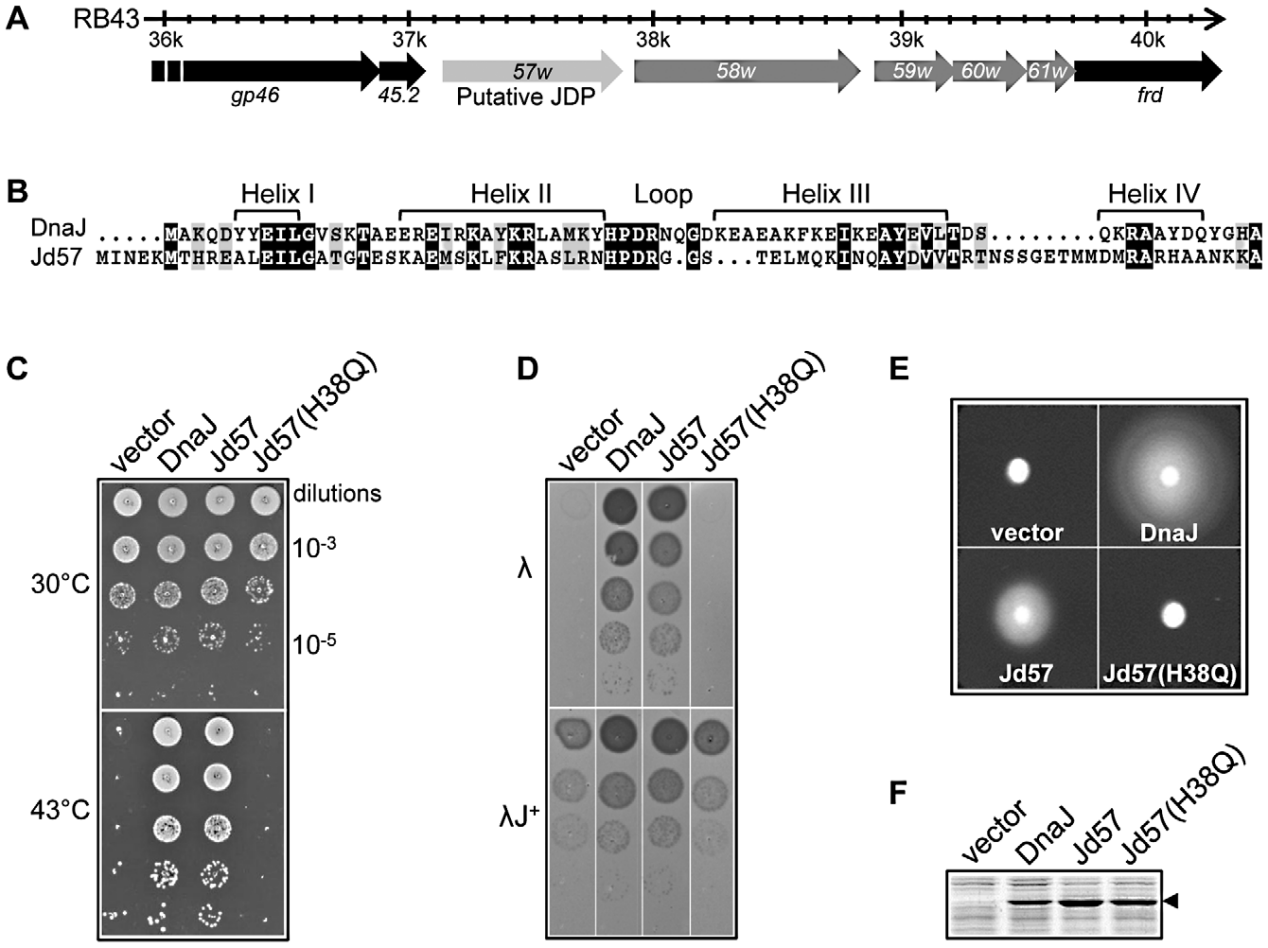
Analysis of the various J-domain chimera phenotypes *in vivo*. (A) The RB43 genome region containing the ORF057w, adapted from http://bacteriophage.bioc.tulane.edu/. Genes in black have orthologs in bacteriophage T4. (B) An alignment of the J-domain primary amino acid sequences using ClustalX. Identical residues are shown in black and conserved substitutions in gray. The limits of α-helical secondary structures in the DnaJ J-domain are also shown (black bars). (C) Complementation of the temperature-sensitive phenotype of the bacterial strain W3110 Δ3 (*dnaJ cbpA djlA* triple mutant) by the various pBAD22-based J-domain chimeras in the presence of 0.01% L-arabinose inducer. Only the origin of the relevant J-domain is shown on top of the figure, the rest of the protein being always that of *E. coli*'s DnaJ. (D) Complementation assay for bacteriophage λ*c*I(λ) plaque formation on strain Δ3 using the pBAD22-based J-domain chimeras in the presence of 0.001% L-arabinose at 30°C. The λ*c*I*dnaJ+* transducing bacteriophage (λJ+) is shown as a positive control. (E) Complementation for bacterial motility assay showing the radial growth of strain Δ3 expressing the pBAD22-based J-domain chimeras in the presence of 0.001% L-arabinose. (F) The relative steady state level of the various DnaJ chimera constructs expressed in strain W3110 Δ3 at 30°C in the presence of 0.1% L-arabinose, following SDS–PAGE of the extracts and staining with Coomassie blue.

The enterobacteriophage RB43 was originally isolated from sewage treatment plants in Long Island [26]. It shares only 40% (115/260) of its genes with T4 and is the representative member of a well-defined group of T4-related bacteriophages, including RB16 and most likely RB42 [27], [28]. The ORFan gene *057w* (Uniprot Q56BZ1) of RB43 is the first gene of a locus of five genes of unknown function absent in bacteriophage T4 (Figure 1A). Interestingly, this gene encodes a putative protein of 237 amino acids, with a N-terminal domain of about 75 amino acids having 63% similarity with the J-domain of the three known *E. coli* DnaJ cochaperones. Specifically, the essential His-Pro-Asp (HDP) tripeptide of the loop connecting helices II and III, as well as key residues from helices II and III of the DnaJ J-domain are well conserved. Nevertheless, the region corresponding to helix IV of DnaJ displays significantly lower sequence conservation (Figure 1B) [29]. With the exception of the four closely related *057w* homologues found in other T4-related bacteriophages, *i.e*., RB16 and RB42, Klebsiella bacteriophage KP15 and Aeromonas bacteriophage 65 [27], no significant sequence similarity with the remaining C-terminal region of the protein was found in databases (Swiss-prot, TrEMBL). Since the C-terminal part of JDP proteins determines localization and/or substrate specificity, the lack of sequence similarity suggested to us that this family of bacteriophage-encoded JDP could recruit the host Hsp70 for specific and potentially novel bacteriophage-related function(s). Surprisingly, in bacteriophage RB16, gene *057w* is fused in-frame with the downstream gene *058w* (Figure 1A; Uniprot Q56BZ0). In this case, the stop codon of *057w* (TAA) is replaced by a glutamate (GAA) at position 238, resulting in a 565 amino acid long fusion protein (Uniprot D9ICB9). A similar fusion is also observed in the less related Klebsiella bacteriophage KP15 (*gp055;* Uniprot D5JF99), but not in RB42 or Aeromonas bacteriophage 65 (http://phage.ggc.edu/blast/blast.html). This suggests that the adjacent *057w* and *058w* genes could somehow cooperate during bacteriophage infection (see below).

We first asked whether the *057w* gene product indeed encodes for a functional JDP, by using domain swapping experiments [30]. Specifically, the J-domain of the *E. coli* DnaJ cochaperone was replaced by the putative J-domain of the *057w* gene product, leading to the formation of the wild-type Jd57-DnaJ chimera. In addition, as a control for an inactive J-domain, we engineered the same chimera with the known inactivating His 38 to Gln (H38Q) substitution in the HPD tripeptide, equivalent to the well-characterized *dnaJ259* mutant allele. This mutation abolishes functional interaction between a J-domain and its cognate Hsp70 [29], [31]. Plasmids expressing various wild-type and chimeric proteins were introduced into an *E. coli* W3110 mutant strain lacking all three endogenous *dnaJ* homologs, namely *dnaJ*, *cbpA* and *djlA* [32]. As a consequence, this strain displays several DnaJ-dependent phenotypes such as temperature-sensitivity, resistance to bacteriophage lambda, and lack of motility [29], [33]. Control experiments with plasmid-encoded wild-type DnaJ confirm that bacterial growth at the nonpermissive temperature of 43°C is indeed DnaJ-dependent (Figure 1C). As anticipated, the Jd57-DnaJ chimera containing the wild-type bacteriophage J-domain efficiently rescues bacterial growth at high temperature, whereas the Jd57(H38Q)-DnaJ mutant chimera does not. In agreement with the high temperature bacterial growth complementation result, bacteriophage λ growth and bacterial motility are also restored by expression of the wild-type Jd57-DnaJ but not by the mutant chimera (Figure 1D and 1E). A control experiment presented in Figure 1F shows that the expression level of the chimeric proteins is comparable under the conditions tested. Taken together, these results demonstrate that the putative *057w* gene product possesses a functional J-domain.

### Bacteriophage-encoded JDP interacts with DnaK *in vivo*

**Figure 2:**
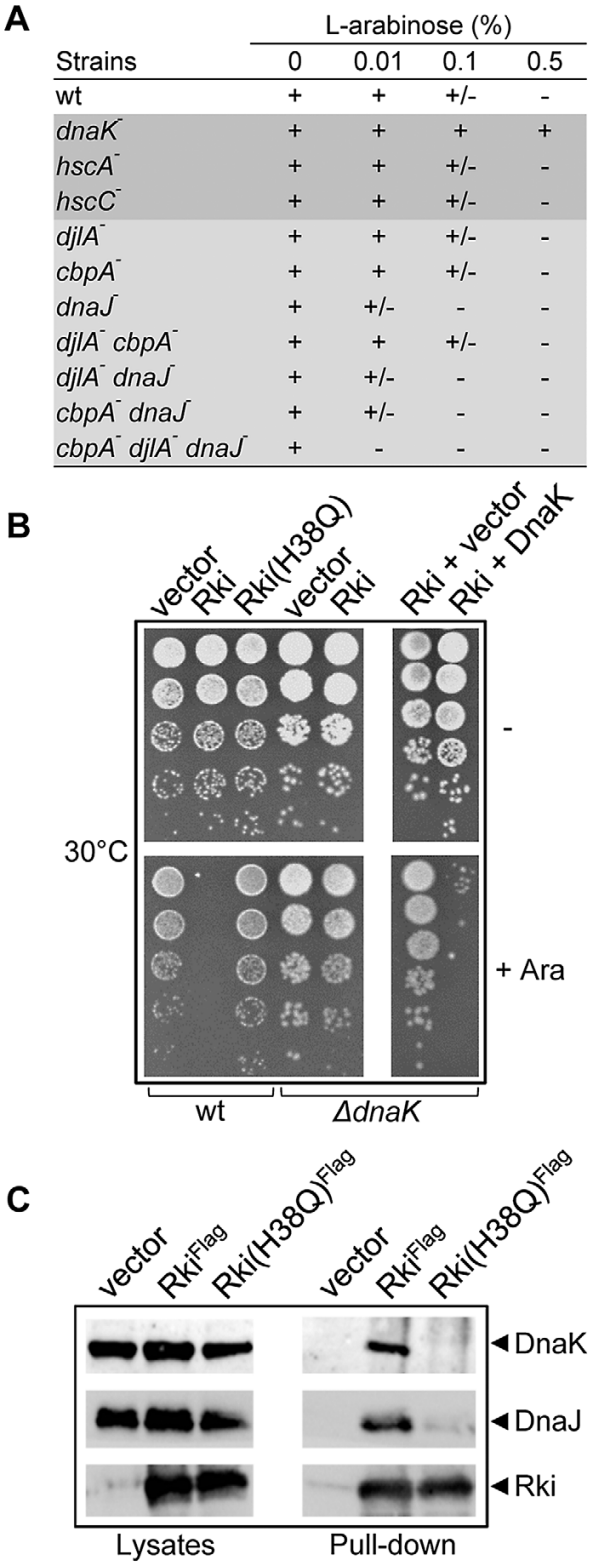
Rki toxicity is DnaK-dependent. (A) Growth of an isogenic set of W3110 derivatives strains expressing the full length bacteriophage-encode JDP on LB ampicillin plates with or without L-arabinose inducer after 18 h incubation at 30°C. The (+) sign indicates no significant difference compared with the empty vector control, (+/−) indicates an approximate efficiency of colony formation of <10^−2^, (−) indicates an efficiency of colony formation of <10^−4^. (B) The arabinose inducible pBAD22-based full-length Rki and Rki(H38Q) constructs were expressed at 30°C in W3110 wild type strain in the absence (−) or in the presence of 1% of L-arabinose inducer (+). The L-arabinose inducible pBAD33-based full-length Rki constructs were expressed with or without inducer at 30°C in Δ*dnaK* (Δ*dnaK*52::Cm^R^) strain alone or in the simultaneous presence of either the compatible empty p29SEN vector or the p29SEN-based *dnaK* gene under the control of its own native promoter. (C) Immunoprecipitation of pBAD24-based Flag-tagged Rki or Rki(H38Q) proteins expressed in *E. coli* wild-type strain. Cell lysates and eluates (Pull-down Flag) revealed by western blot analyses using anti-Rki, anti-DnaJ and anti-DnaK antibodies.

Since similarity between the putative bacteriophage-encoded JDP and the other DnaJ family members is restricted to the J-domain, by convention this protein belongs to the type III group, like known JDPs of eukaryotic viruses [22]. We next asked whether the full length protein encoded by *057w* displays some DnaJ function *in vivo*. However, multiple attempts to clone *057w* under the control of its native promoter were unsuccessful, even when a pSC101 low copy number plasmid was used as a cloning vector. Finally, *057w* was successfully cloned under the control of the tightly regulated P*araBAD* promoter in the presence of glucose to minimize basal transcription levels and thus avoid toxicity. This plasmid construct was then tested for complementation of the temperature-sensitive phenotype of the triple *dnaJ cbpA djlA* mutant. As expected, expression of the full length bacteriophage JDP is highly toxic in the presence of L-arabinose inducer and is thus not capable of replacing the *E. coli* DnaJ protein (Figure 2A). To investigate whether the severe toxicity is DnaK-dependent, we then expressed the bacteriophage JDP in the single *dnaK*, *hscA* or *hscC* mutants, the three Hsp70-encoding genes of *E. coli* [7], and monitored its effect on bacterial growth. As in the wild-type strain, expression of the bacteriophage JDP exhibits a strong toxic effect in both *hscA* and *hscC* mutants. In sharp contrast, the JDP displays no toxicity when expressed in the single *dnaK* mutant (Figure 2A and 2B), thus indicating that its toxicity is DnaK-dependent. As expected, the toxicity is restored when DnaK is co-expressed from a plasmid (Figure 2B).

Next, we showed that overexpression of the bacteriophage JDP harboring the H38Q inactivating mutation in its J-domain does not result in toxicity when expressed in the wild-type *E. coli* strain, thus demonstrating that the DnaK-dependent toxicity also requires a functional J-domain (Figure 2B). Note that both the wild-type and H38Q JDP mutants showed comparable steady state expression levels (Figure S1). Toxicity of the bacteriophage JDP was exacerbated in the sole absence of DnaJ, the main cochaperone of DnaK *in vivo* (and to a lesser extent in the presence of CbpA and DjlA), suggesting that the bacteriophage protein may compete with DnaJ for binding to DnaK during bacteriophage infection (Figure 2A). Since the bacteriophage JDP and DnaK genetically interact, the gene *057w* was named *rki* for *R*B43 Dna*K*-*i*nteractor.

The contribution of the uncharacterized C-terminal domain of Rki to the DnaK-dependent toxicity remains unknown. Based on the predicted secondary structure of Rki (and on the partial chymotrypsin proteolysis of purified Rki protein described below and in Figure S2), we engineered a C-terminal deletion of Rki, after amino acid Met159, within a predicted random coil region located approximately halfway through the putative C-terminal domain. The resulting Rki(1–159) construct was tested for both, its toxicity and its ability to replace the *E. coli* DnaJ cochaperone during bacterial growth at non-permissive temperature. Strikingly, robust overexpression of Rki(1–159) exhibits no toxicity at all, thus indicating that the C-terminal and the J-domain of Rki act in concert to exert toxicity. In addition, Rki(1–159) is also able to partially replace DnaJ as a functional DnaK cochaperone *in vivo*, even at the stringent temperature of 43°C (Figure S1). These results reveal that the DnaK-dependent toxic effect of Rki relies on both a functional J-domain and the C-terminal domain of unknown function.

We next asked whether Rki and DnaK could indeed physically interact *in vivo*. To do so, an N-terminal Flag-tagged version of Rki was expressed in *E. coli* and used as bait in pull-down experiments. The results shown in Figure 2C clearly demonstrate that indeed Rki and DnaK form a complex. The fact that the H38Q substitution in Rki affects interaction with DnaK in this assay confirms that the interaction necessitates a functional J-domain (Figure 2C). In addition, DnaJ participates in the complex with Rki and DnaK (Figure 2C). However, as with DnaK, the complex between DnaJ and Rki is disrupted by the presence of the H38Q substitution in the Rki J-domain, thus indicating that the presence of DnaJ is DnaK-dependent and is not simply due to the formation of mixed oligomers between Rki and DnaJ. Taken together, these data demonstrate that Rki possesses a functional J-domain, which enables it to functionally interact with the host multifunctional DnaK chaperones *in vivo*.

### Rki exhibits no generic (co)chaperone function *in vitro*

To further explore Rki functions *in vitro*, we purified both the wild-type Rki and Rki(H38Q) mutant proteins. SEC-MALLS experiments performed with purified Rki shows that Rki elutes as a single peak with an average molecular mass of 31.5 kDa (Figure S2; Text S1). This is in good agreement with the theoretical molecular mass of 29.15 kDa demonstrating that in contrast to the three *E. coli* J-domain cochaperones DnaJ, CbpA and DjlA, Rki is almost exclusively monomeric in solution. In addition, partial α-Chymotrypsin proteolysis followed by N-terminal sequencing of purified Rki indicates a two domain structure composed of the N-terminal J-domain (residues 2 to 70), a short putative linker region (residues 71 to 75) and a larger C-terminal domain (residues 76 to 237; Figure S2).

**Figure 3:**
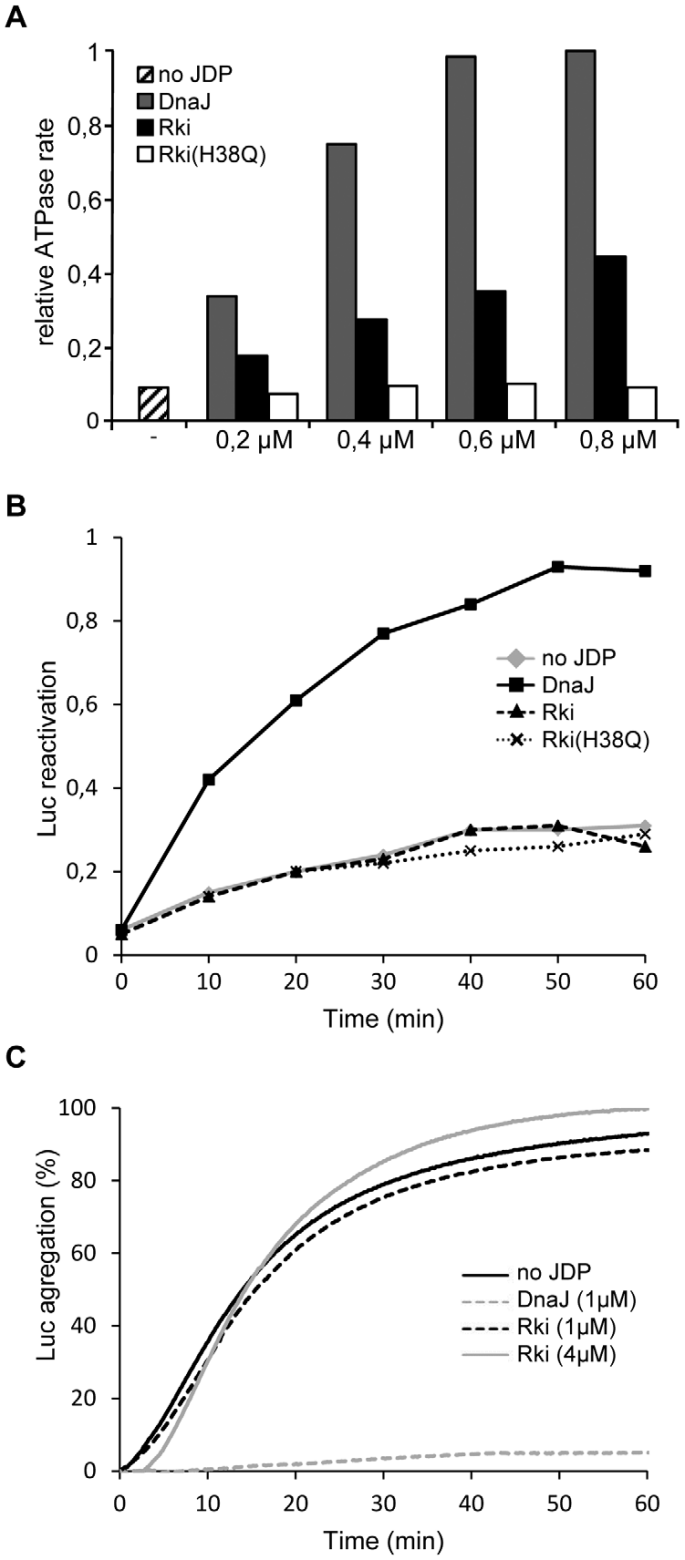
Rki cochaperone functions *in vitro*. (A) Stimulation of DnaK ATPase activity under steady state conditions. DnaK(1 µM) and GrpE (0.5 µM) in the absence or in the presence of DnaJ, Rki or Rki(H38Q) at the indicated concentrations. The percentage hydrolyzed ATP/min is plotted as a function of the final DnaJ concentration used in the reaction mix. (B) Refolding chemically-denatured firefly luciferase (125 nM) by DnaK (500 nM) and GrpE (125 nM) in the presence of 125 nM of either DnaJ, Rki or Rki(H38Q) as indicated. The values of luciferase refolding were normalized to the maximal value obtained with that of wild type DnaJ. (C) Luciferase aggregation protection assay. A representative plot of a luciferase aggregation protection assay is shown with chemically-denatured luciferase (1 µM) alone (no JDP), or in the presence of DnaJ (1 µM), Rki (1 µM or 4 µM). Optical densities were measured at 320 nm and the percentage values were normalized to the luciferase aggregation obtained in the absence of added chaperones.

Purified Rki and Rki(H38Q) proteins were then tested for their ability to stimulate DnaK's ATPase activity *in vitro* under steady state condition, as described [33]. The results presented in Figure 3A show that Rki wild-type indeed stimulates DnaK ATPase, although less efficiently than does DnaJ. In contrast, Rki(H38Q) harboring the inactivating mutation the J-domain does not show any stimulation, thus indicating that Rki is capable of stimulating DnaK ATPase activity in a J-domain dependent manner. This result is in agreement with the domain swapping experiments shown in Figure 1 and further demonstrates that Rki possesses a *bona fide* JDP.

We next asked whether Rki could assist DnaK in the refolding of the chemically denatured luciferase substrate. This assay is dependent on both a functional J-domain and a capacity to bind to and deliver an unfolded substrate to DnaK. A representative kinetic analysis of luciferase refolding in the presence of Rki, Rki(H38Q) or DnaJ is shown in Figure 3B. As expected, DnaJ efficiently stimulates DnaK-mediated refolding of luciferase. In contrast, both Rki and Rki(H38Q) do not stimulate DnaK's reactivation activity, even when Rki concentration was increased 2-fold above that of DnaJ. These results strongly suggest that although Rki interacts with DnaK both *in vivo* and *in vitro*, it does not possess a DnaJ-like, generic cochaperone function. This behavior is in sharp contrast to that of DnaJ, CbpA and DjlA [7].

It is known that DnaJ possesses intrinsic chaperone function, as it can bind unfolded substrate and efficiently prevents its aggregation [16]. The inability of Rki to support DnaK-mediated reactivation suggests that it may not efficiently bind denatured luciferase. Indeed, the results presented in Figure 3C clearly show that Rki alone does not prevent the aggregation of chemically denatured luciferase, even at a much higher cochaperone/substrate ratio, thus indicating, once more, that Rki displays no apparent generic chaperone function.

In summary, the above results demonstrate that Rki specifically interacts with DnaK in a J-domain dependent manner. However, in sharp contrast with DnaJ, CbpA or DjlA, Rki is not capable of assisting DnaK as a generic cochaperone *in vitro* (Figure 3B) or throughout its multiple cellular tasks, as judged by its inability to replace DnaJ functions *in vivo* (Figure 2).

### Rki is expressed early after infection by the bacteriophage RB43

**Figure 4:**
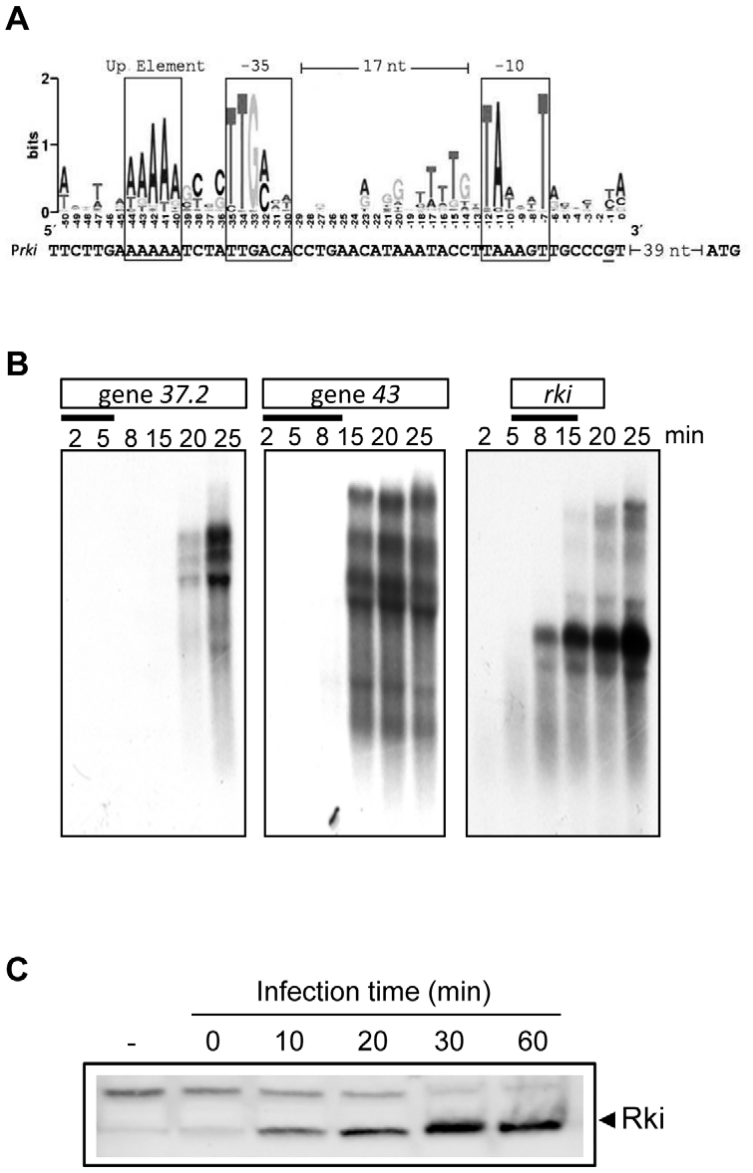
Rki is transcribed early during bacteriophage infection. (A) Early promoter mapping. The WebLogo for RB43 early promoter was determined as described in Nolan *et al*., 2006. The G nucleotide underlined is the putative transcription start site. Putative up elements and the −10 and −35 region are boxed. (B) Northern blot analysis showing transcription of *rki* and two control genes known to be transcribed in the early (*g43*) or late (*g37.2*) phase of infection. (C) Western blot analysis of whole cell extracts prepared from W3110 cells non-infected (−) or infected with RB43 at a MOI ∼10, during 10, 20, 30, or 60 min at 30°C and revealed using an anti-Rki rabbit antibody.

To investigate a possible Rki function *in vivo*, we analyzed the presence of a putative bacteriophage promoter as well as the occurrence of *rki* transcripts during the course of RB43 infection. The putative *rki* gene promoter was identified and analyzed by a comparison with the consensus promoter described by Nolan *et al*. [34]. The putative *rki* promoter turns out to be very similar to the consensus RB43 early promoters with TAAAGT and TTGACA boxes located at −10 and −35 positions, respectively, and a consensus up element (Figure 4A). Subsequently, we performed northern blot analysis using as controls two other genes known to be transcribed in the early (*g43*) or late (*g37.2*) phase of infection. As shown in Figure 4B, the *rki* transcript appears early, at 5 to 8 min following infection. These data are in agreement with the predicted presence of the *rki* early promoter. Finally, we asked whether the *rki* gene product is actually expressed during infection, by using a polyclonal antibody raised against Rki. The western blot analysis results presented in Figure 4C clearly show that, indeed, the Rki protein is expressed during the early phase of RB43 infection.

### Rki facilitates bacteriophage growth in the presence of DnaK

**Figure 5:**
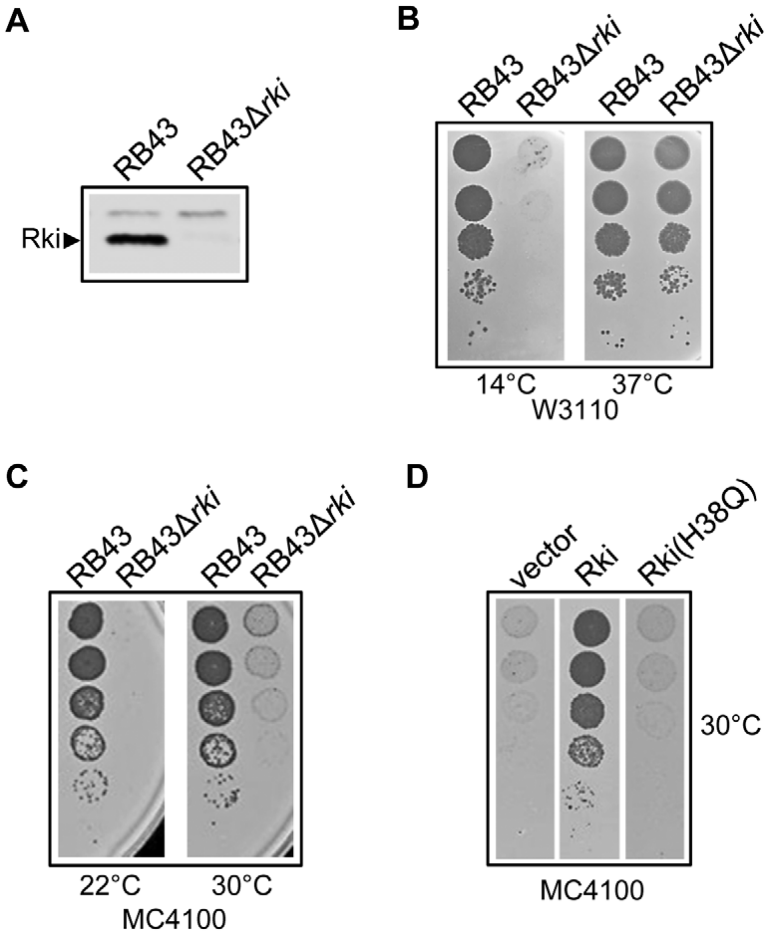
The RB43Δ*rki* growth phenotype. (A) Western Blot analysis using anti-Rki antibody and performed on whole cell extracts of W3110 cells infected by either RB43 or RB43Δ*rki* for 30 min at 30°C. (B and C) Bacteriophages RB43 and RB43Δ*rki* plaque-forming abilities on *E. coli* strain W3110 (B) or MC4100 (C). H-Top plates were incubated either overnight at 30° and 43°C or for 2 days at 22° or 14°C. (D) Complementation assay for RB43Δ*rki* plaque formation on MC4100 strain transformed with either the plasmid p29SEN empty vector, p29SEN-Rki or p29SEN-Rki(H38Q) in the absence of IPTG inducer.

It is known that at least some of the bacteriophage T4 proteins synthesized immediately following infection confer selective advantages to bacteriophages under specific environmental conditions, thus facilitating the timely progression from host to bacteriophage metabolism [35]. To examine such a possible role for Rki *in vivo*, we first engineered a deletion/replacement of *rki* by the *gfp* gene (encoding green fluorescence protein) by homologous recombination into RB43 genome. Because RB43 wild-type grows better on a *dnaK* mutant than on the isogenic wild-type strain in certain *E. coli* host backgrounds (see below) and because Rki toxicity is strictly DnaK-dependent, the Δ*rki::gfp* mutant was isolated on a *dnaK* mutant host (see the Materials and Methods section for details). Following recombination into the bacteriophage genome and PCR verification of the correct deletion/replacement, the absence of the Rki protein during bacteriophage infection at 30°C was confirmed by western blot analysis using polyclonal anti-Rki antibody (Figure 5A).

The RB43Δ*rki* mutant and its isogenic parent were then tested for their ability to form plaques on wild-type *E. coli* hosts at various temperatures. Note that in sharp contrast with bacteriophage T4, RB43 wild-type grows fairly well below 16°C and very poorly above 37°C (Figure S3). We found no significant difference at 37°C between the wild-type and RB43Δ*rki* mutant when grown on the *E. coli* W3110 strain background. However, growth of RB43Δ*rki* mutant was severely compromized at 14°C compared to the RB43 wild-type parent (Figure 5B). The effect of the *rki* mutation on RB43 growth was significantly more severe when *E. coli* MC4100 was used as the host strain, as judged by the reduced plaque-forming ability of RB43Δ*rki* mutant already observed at 30°C (Figure 5C). To ensure that the phenotype was Rki-specific, we performed complementation experiments using Rki expressed from a plasmid under the control of an inducible promoter. The results presented in Figure 5D and Figure S4, for MC4100 and W3110 strains respectively, show that the mutant growth defects are indeed due to the lack of Rki function. Taken together, these *in vivo* results indicate that although *rki* is not an absolutely essential gene, its presence confers a significant advantage to RB43 during infection, especially at more stringent temperatures (*i.e*., cold) and can vary significantly depending on the particular *E. coli* host being infected.

Next, we asked whether the phenotype of the Δ*rki* mutant is indeed due to the lack of functional interaction with the host DnaK chaperone. To do so, we expressed the Rki(H38Q) mutant from a plasmid and tested its ability to complement the lack of Rki during bacteriophage infection. The results obtained in both the MC4100 (Figure 5D) and W3110 (Figure S4) strain backgrounds clearly show that the J-domain mutant is not capable of complementing for the lack of Rki function at the non-permissive temperature of growth. In this case, expression of plasmid-encoded Rki(H38Q) was comparable to that of Rki wild-type (Figure S4). This result clearly demonstrates that Rki acts through a functional interaction with the DnaK chaperone *in vivo* during infection.

### A host mutation in *dnaK* suppresses the need for Rki

The above results suggest that early during infection, Rki may recruit the DnaK chaperone function to directly facilitate various bacteriophage processes, such as transcription, DNA replication or protein folding. Yet, in sharp contrast with bacteriophages λ, P1 and P2, T4 does not require the DnaK/DnaJ/GrpE chaperone machine for its DNA replication on an *E. coli* host [7], [36]–[38]. Alternatively, Rki could inhibit a DnaK cellular function(s) detrimental to its proliferation, as it has been proposed for the host Hsp40 chaperone, which inhibits hepatitis B virus replication and capsule assembly [39].

**Figure 6:**
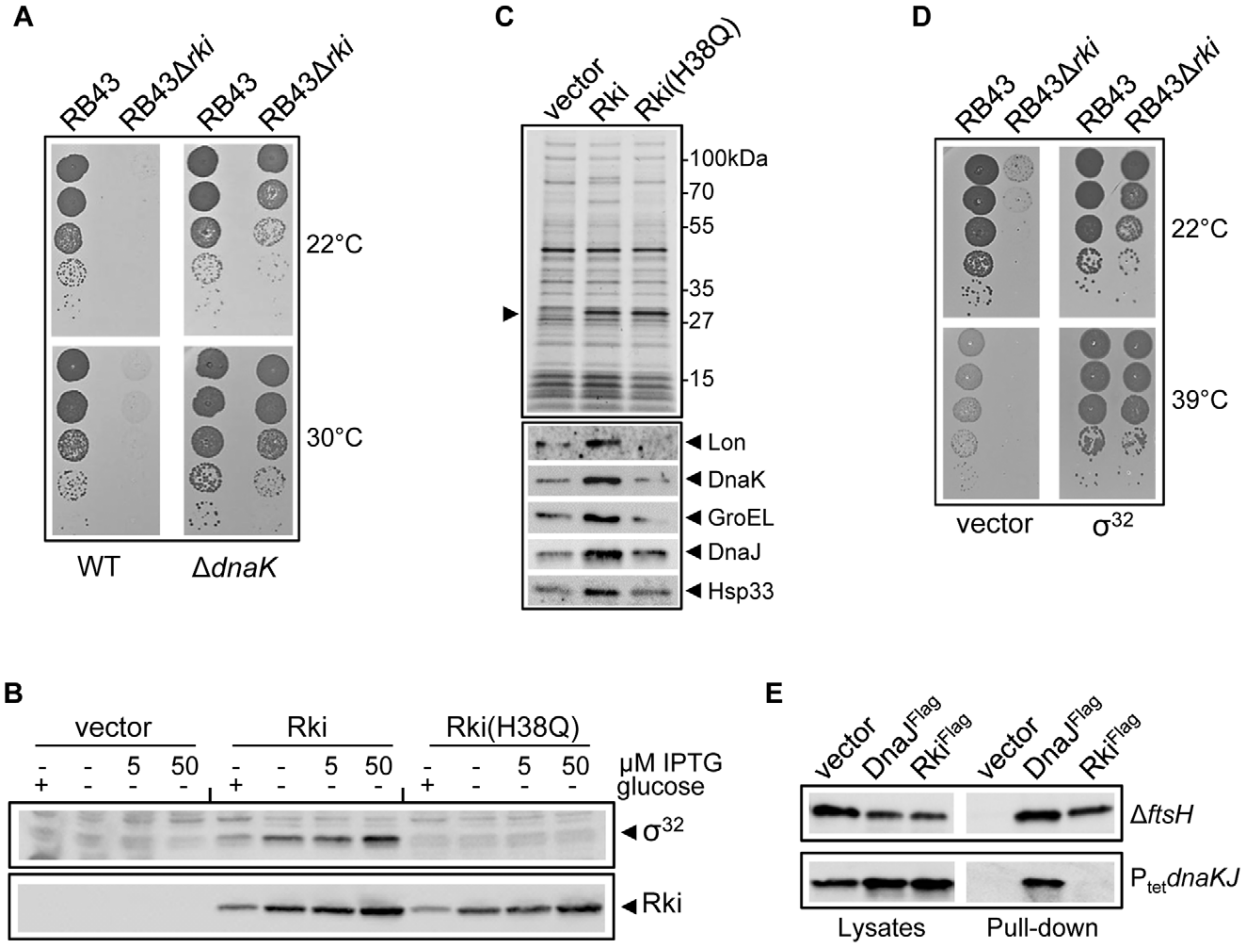
Rki stabilizes the heat-shock factor σ^32^ *in vivo*. (A) Bacteriophage RB43 and RB43Δ*rki* lysates were serially diluted and spotted on *E. coli* MC4100 and on its MC4100 Δ*dnaK52 sidB1* mutant derivative. Plates were incubated either overnight at 30°C or for 2 days at 22°C. (B) Western blot analysis (probed with anti-Rki and anti-σ^32^) of whole cell extracts from strain MC4100 transformed with p29SEN empty vector, p29SEN-Rki or p29SEN-Rki(H38Q) grown in the presence of the indicated IPTG or 0.2% glucose (+) inducer. (C) Whole cell extracts of strain MC4100 transformed with either the p29SEN empty vector, p29SEN-Rki or p29SEN-Rki(H38Q) and grown in the presence of 50 µM IPTG were separated by SDS-PAGE and stained with Coomassie blue. The same samples were analyzed by western bolt analysis using antibodies raised against the indicated chaperones or the Lon protease. (D) Bacteriophage RB43 and RB43Δ*rki* lysates were serially diluted and spotted on *E. coli* MC4100 transformed with p29SEN empty vector, p29SEN-RpoH (σ^32^). Plates were incubated overnight at 39°C or 22°C. (E) *In vivo* coimmunoprecipitation of pBAD33-based Flag-tagged DnaJ and Rki proteins expressed in the *E. coli* AR3291(Δ*ftsH*) or FA1195 (P_tet_ *dnaKJ*) mutant strains co-transformed with p29SEN-RpoH at 30°C in the presence of 0.5% L-arabinose and 50 µM IPTG. Cell lysates and eluates (Pull-down) were revealed following western blot analysis using anti-σ^32^ antibody.

To investigate this possibility, we first asked whether a mutation in *dnaK* restores growth to RB43Δ*rki*. We used the MC4100 strain, which does not efficiently propagate RB43Δ*rki* even at 30°C, a permissive temperature for a *dnaK* mutation, as a suitable host for such experiments (Figure 5C) [40]. We compared the ability of RB43 wild-type and RB43Δ*rki* to form plaques on MC4100 and on its Δ*dnaK52*::Cm^R^ *sidB1* (BB1553) isogenic mutant derivative [40]. Note that the *dnaK* mutant strain BB1553 carries the *sidB1* suppressor mutation in *rpoH*, allowing the cells to grow stably at 30°C [40]. As suspected, we found that the absence of DnaK efficiently suppresses the growth defect of RB43Δ*rki* mutant (Figure 6A). These results are in strong agreement with the DnaK-dependent toxicity of Rki and suggest that Rki, via its functional J-domain, could counteract some putative antagonistic function(s) of DnaK on bacteriophage RB43 growth.

### Rki expression stabilizes the heat-shock factor σ^32^ and induces heat-shock proteins

One of the main cellular functions of the DnaK/DnaJ/GrpE chaperone machine in *E. coli* is to control the entire σ^32^-dependent heat-shock response. Under normal conditions, it is known that DnaK and DnaJ can bind and target the heat-shock factor σ^32^ for degradation by the membrane-anchored FtsH protease, thus autoregulating their own synthesis and limiting that of the other σ^32^-dependent heat-shock proteins (HSPs) [41]. Following a heat stress, the DnaK chaperone is rapidly titrated away from σ^32^ by being recruited to unfolded and aggregated proteins, thus resulting in the stabilization of σ^32^. In turn, stabilized σ^32^ binds to the RNA polymerase core leading to the transcription and induced transcription of more than one hundred *HSP* genes [42], [43]. In agreement with such DnaK function, the deletion of *dnaK* leads to a 3–4 fold increase in the HSPs steady state levels, including the major stress chaperones (*e.g*., GroEL, HtpG, ClpB, IbpA/B) and proteases (*e.g*., FtsH, Lon, ClpXP, HslUV).

It is known that following infection with various eukaryotic viruses the synthesis of HSPs, including the chaperones Hsp27, Hsp70, Hsp40 and Hsp90 is induced (review in [20]). In some cases, the increased level of HSPs directly helps viral replication as it has been observed with the SV40, HIV-1 or CELO viruses [44]–[46]. Recently, Rawat and Mitra have shown that in human cell lines, the heat-shock factor 1 (HSF1), the major eukaryotic transcription factor that regulates transcription of the *HSP* genes in response to stress, is specifically induced during HIV-1 infection to directly drive viral gene expression and promote its own replication [44].

Taken all of the above observations together, we reasoned that immediately after infection, Rki may bind DnaK, thus triggering σ^32^ release and/or may somehow prevent its degradation by the FtsH protease. We first tested whether Rki expressed from a plasmid affects the levels of σ^32^ in the presence of DnaK. The results shown in Figure 6B demonstrate that indeed, expression of Rki rapidly leads to an increase in the endogenous σ^32^ levels. In sharp contrast, expression of the inactive Rki(H38Q) J-domain mutant does not affect σ^32^ levels, indicating that this process is DnaK-dependent. As expected, the level of HSPs was also concomitantly increased (Figure 6C). Remarkably, further *in vivo* experiment revealed that co-overexpression of plasmid-encoded σ^32^ exacerbates Rki toxicity (Figure S5). This result strengthens the genetic link between Rki and σ^32^ and is in agreement with previous works demonstrating that high endogenous levels of σ^32^ are deleterious for *E. coli* at 30°C in the absence of a functional DnaK, possibly due to inappropriately high levels of HSPs [40], [47]. We next asked whether the increased levels of endogenous σ^32^ in a wild-type *E. coli* background could help growth of the RB43Δ*rki* mutant. The results presented in Figure 6D clearly show that indeed, expression of σ^32^ from a low-copy number plasmid fully suppresses the growth defect of RB43Δ*rki*, even at the stringent temperatures of 22° and 39°C.

The DnaK-dependent stabilization of σ^32^ by Rki suggests that Rki either inhibits DnaK, thus indirectly preventing σ^32^ transfer to FtsH at the membrane, or directly binds σ^32^ in complex with DnaK and prevents its degradation. To begin to answer such questions, we co-expressed a Flag-tagged Rki and wild-type σ^32^ in an *ftsH* mutant strain (to avoid degradation of σ^32^) and performed *in vivo* pull-down experiments as described in Figure 2C. As a control, the same experiment was performed simultaneously with either a Flag-tagged DnaJ or the pBAD33 empty vector. The result presented in Figure 6E shows that as observed for DnaJ, Rki binds σ^32^ *in vivo* in the presence of DnaK. To investigate whether Rki binding to σ^32^ is dependent on DnaK, we next performed the same *in vivo* pull-down experiments using a DnaK depletion strain, in which chromosomally-encoded DnaK is under the control of a Tet-inducible promoter. Under the growth conditions tested, DnaK is barely detectable by western blot in the absence of anhydrotetracycline when compared to the isogenic wild-type strain (Figure S6). The results presented in Figure 6E clearly show that efficient binding of Rki to σ^32^ indeed depends on the presence of DnaK (Figure 6E), thus suggesting that Rki could stabilize σ^32^ by acting directly on the DnaK-σ^32^ complex.

How does stabilization of σ^32^ by Rki help RB43 growth? Clearly, the increased levels of σ^32^ rapidly results in much higher intracellular levels of all HSPs, including GroEL which is absolutely essential for the proper folding of the bacteriophage RB43 major capsid protein Gp23 [48]. In agreement with this, Wiberg *et al.* (1988) showed that indeed the progeny yield of bacteriophage T4 increases dramatically when HSP synthesis is induced prior to bacteriophage infection, as does overexpression of the GroES/GroEL chaperone from a plasmid. However, in the case of Rki, induction of HSPs would occur shortly after infection, well before the synthesis of the capsid Gp23 protein. In sharp contrast with the full suppression exhibited by plasmid-encoded Rki and σ^32^, we found that overexpression of GroESL only weakly suppresses the growth defect of RB43Δ*rki*, as judged by the turbid plaques of RB43Δ*rki* observed only at the less stringent temperature of 30°C on the MC4100 background (Figure S6). Nevertheless, in the context of infection, even a modest increase in GroEL levels could translate into an increase in Gp23 folding, resulting in a slight increase in bacteriophage production. Outside the laboratory, these seemingly minor increases would result in a small but significant selective advantage for maintaining *rki* in the genome.

An alternative hypothesis is that stabilization of the heat-shock factor σ^32^ immediately after infection could directly help transcription of RB43 middle and/or late genes. Despite the fact that T4 encodes its own sigma factor gp55 for late transcription, it has been shown that a temperature upshift (from 30° to 42°C) performed a few minutes after infection by T4 dramatically affects transcription of late genes in the absence of σ^32^, by an as yet unknown mechanism [49]. Such a mechanism involving σ^32^ could thus facilitate RB43 late gene expression under nonheat-shock conditions. In T4, it is known that activation of transcription from middle promoters requires the host RNA polymerase and σ^70^, as well as the two bacteriophage proteins MotA and AsiA [35]. Intriguingly, an in-depth comparative analysis of RB43 and T4 promoter regions neither detected middle promoter consensus sequences nor identified a *motA* ortholog in RB43, thus suggesting a very different mechanism [34].

### Concluding remarks

This work shows for the first time that bacteriophages can encode functional J-domain proteins capable of hijacking the host Hsp70 chaperone to facilitate viral proliferation. Our results show that, at least in the case of Rki, interaction with the host DnaK prevents degradation of the heat-shock factor σ^32^ via an unknown mechanism, thus conferring a selective advantage for RB43 under certain circumstances.

As stated above, in bacteriophage RB16 the *rki* gene is fused with the downstream ORF*058w*, due to a single substitution in the stop codon of *rki*. The presence of the Rki-58 fusion protein, named Rki16, during infection by RB16 was confirmed by western blot analysis, thus excluding the possibility of a DNA sequencing artifact (Figure S7). In addition, we found that overexpression of plasmid-encoded Rki16 fusion protein stabilizes σ^32^ and complements the RB43Δ*rki* growth-sensitive phenotype, albeit considerably less efficiently than Rki. In agreement with this, the Rki-58 fusion protein is considerably less toxic than Rki (Figure S7). To date, nothing is known about the function of RB43 ORF*058w*, whose product possesses a weak similarity with an uncharacterized conserved domain PTZ00121 at its C-terminus (http://www.ncbi.nlm.nih.gov). Yet, in the case of Rki, co-overexpression of the ORF*058w* gene product does not affect either the stabilization of σ^32^ or Rki toxicity (Figure S7). This indicates that within the limit of our experimental conditions, ORF*058w* does not significantly influence Rki function in RB43.

Interestingly, in addition to its J-domain protein Rki, the bacteriophage RB43 possesses two other uncharacterized small ORFan genes, namely ORF*179c* (61 amino acid residue gene product; Uniprot Q56BL9) and ORF*191c* (106 amino acid residue gene product; Uniprot Q56BK7), whose gene products displays significant sequence similarity with the conserved zinc-binding domain of DnaJ, known to be critical for both substrate binding and activation of the DnaK chaperone cycle [50]. It is intriguing that RB43 potentially expresses several proteins that display homology with distinct domains important for DnaJ cochaperone function. One attractive hypothesis is that multiple DnaJ-like bacteriophage proteins could act in concert to hijack (or inhibit) the host DnaK/DnaJ/GrpE chaperone machine in order to facilitate bacteriophage proliferation under different environmental conditions.

## Materials and Methods

### Bacterial strains and bacteriophages

Genetic experiments were carried out in *E. coli* K-12 MC4100 or W3110 strains. Strains MC4100 and BB1553 [40], and AR3291 (W3110 *sfhC21 zad220*::Tn*10* Δ*ftsH3*::Kan^R^; [6]) have been described. The strain FA1195 P_tet_ *dnaKJ* is an MG1655 derivative in which the endogenous *dnaKdnaJ* promoter is replaced by the tetracycline promoter P_tet_, together with the upstream *terR* repressor. In this case, expression of DnaK is dependent on the presence of anhydrotetracycline (Frederic Angles, laboratory collection). Mutations were moved in different genetic backgrounds using bacteriophage P1-mediated transduction at 30°C. To construct the single, double or triple JDP mutants in the W3110 strain background, the Δ^3^ strain (MC4100 *dnaJ*::Tn*10*-42, Δ*cbpA*::kan^R^, Δ*djlA*::Ωspc^R^; [33]) was used as donor. The Δ*dnaK*52::Cm^R^ [2], Δ*hscC*::kan^R^ (JWK0645; Keio Collection) and Δ*hscA*::kan^R^ (JWK2510; Keio Collection) mutant alleles have been described. Bacteriophages RB43 [26], λ*c*I, λ*c*I*dnaJ* ^+^ and P1 (laboratory collection) were maintained on W3110 at 30°C.

The RB43Δ*rki::gfp* deletion/replacement mutant was constructed as follows. The 717 bp long *gfp* gene was first amplified using primers RBGFP1 (5′- GAACGGAAAATGAGTAAAGGAGAAGAAC) and RNGFP3 (5′-CATTACCGCTAATTTATTTGTAGAGCTCATCC). Thr 1212 bp region upstream *rki* was amplified using primers RB1 (5′-GCAGGATCCCTGGTGCAGACCGAACGG) and RBGFP4 (5′-CTTTACTCATTTTCCGTTCCTCAAAATAAAAG), and the 835 bp region downstream *rki* was amplified using primers RBGFP2 (5′-CTACAAATAAATTAGCGGTAATGATATCTATG) and RB3 (5′-CCCAAGCTTGGGCATGAGCCTTATCAACTGCTG). The three PCR fragments were assembled by the two-step fusion PCR method, resulting in a 2764 bp long fragment containing the *gfp* gene flanked by both the upstream and downstream genomic regions of *rki*. This fragment was then digested with *Hind*III and ligated into plasmid pMPMA6Ω previously digested with *Eco*RV and *Hind*III. Next, the *E. coli* B178 strain transformed with the resulting plasmid was grown to mid-log phase at 30°C in LB supplemented with ampicillin and 200 µl of the culture was then infected with 10^6^ RB43 bacteriophages for 20 min at 20°C. Eight ml of LB amp were added and the culture was incubated at 37°C for 3 h with shaking until lysis occurred. Next, 100 µl of mid-log phase culture of B178 *dnaK103* mutant strain was mixed with the bacteriophage lysate from above to obtain about 400 pfu per plate following overnight incubation at 30°C. Plaques were then transferred to nitrocellulose filters by Benton Davies method and DNA was bound with Stratalinker. The prehybridation took place at 68°C for 3 h and hybridation was carried out overnight at 68°C. These two steps were carried on with *gfp* DNA fragment labeled with Dig and the reaction tubes were boiled for 10 min. Then, the filters were washed at 65°C first in 2× SSC, 0.1% SDS and then in 0.1× SSC, 0.1% SDS before they were incubated with anti-Dig alkaline phosphate and revealed with NTB + BIPC. The genomes of both RB43 wild-type (accession HE858210) and RB43Δ*rki::gfp* mutant (accession HE981739) used in this study were sequenced using the NGS/Illumina method (LGC Genomics). Analysis of the wild-type genome revealed that the sequence (with approximately 99% coverage) of the RB43 bacteriophage used in this study differs from the published RB43 genome sequence by at least 107 nucleotides (http://phage.ggc.edu/). Apart from the Δ*rki::gfp* deletion/replacement, 106 of these nucleotide differences were common to both RB43 wild-type and RB43Δ*rki::gfp* mutant, whereas one mutation was different in the two bacteriophages but affected the same codon. This mutation was located in the hypervariable region of a putative adhesin gene *38* and corresponds to single nucleotide changes, CAA(Gln100) to AAA(Lys) for the wild-type and to CGA(Arg) for the RB43Δ*rki::gfp* mutant. Changes for lysine or arginine residues at this position in gp38 from bacteriophages RB42 and RB43 are known to facilitate recognition of the *E. coli* K-12 hosts [28]. Moreover, one mutation was found only in the RB43Δ*rki::gfp* mutant. This mutation corresponds to a single nucleotide change GCT(Ala5) to CCT(Pro) in gene *62* encoding for one subunit of the clamp-loader (Gp44/Gp62) involved in T4 DNA replication and transcription of late genes [51]. Whether these mutations are linked to the simultaneous deletion of *rki* is unknown. Arguing against this possibility, the mutations were not present in another, independent, cold-sensitive RB43Δ*rki::gfp* bacteriophage isolate. In addition, overexpression of the wild-type Gp62 from a plasmid did not rescue the cold-sensitive phenotype of either RB43Δ*rki* (Elsa Perrody, unpublished data).

### Plasmid constructs

Plasmids pBAD22, pBAD33 and pBAD24 (Guzman *et al*., 1995), p29SEN (Genevaux *et al*., 2004), pWKG90(pBAD22-DnaJ) and pWKG90KPN(pBAD22-DnaJ-H71T) [30], pGPPK [33] have been described previously.

To construct plasmid pBAD22-DnaJ^Flag^, containing DnaJ with the N-terminal Flag tag “MASDYKDDDDKSG”, the *dnaJ* gene was PCR-amplified using primers dnaJ-flagfor (5′-GCGAATTCATGGCAAGCGACTACAAAGATGACGACGATAAAAGCGGCATGGCTAAGCAAGATTATTACG) and dnaJrev (5′-GCAAGCTTGCATGCTTAGCGGGTCAGGTCGTCAA), and pWKG90 DNA as template. The resulting PCR fragment was digested with *Eco*RI and *Sph*I and ligated into pBAD22 previously digested with the same enzymes. Plasmid pBAD33-DnaJ^Flag^ was then constructed by subcloning of the *Eco*RV/*Sph*I DnaJFlag fragment from pBAD22-DnaJ^Flag^ into *Eco*RV/*Sph*I pBAD33.

Plasmid pBAD22-Rki was constructed as follows. The 714 bp long *rki* gene was PCR amplified using primers RB43DnaJfor (GCGAATTCATGATTAACGAAAAAATGACA) and RB43DnaJrev (GCAGATCTAAGCTTTATGCGTCTAAGTGCTTGCG), digested with *Eco*RI and *Bgl*II and ligated into pBAD22 previously digested with the same enzymes. The pBAD22*-rkiH38Q* plasmid was constructed by the two-step PCR method using mutant primers H38Qfor (5′-CTCTGCGTAATCAGCCCGATCGTGG-3′) and H38Qrev (5′-CCACGATCGGGCTGATTACGCAGAG-3′). To construct pBAD33-Rki, the *rki* gene was subcloned from pBAD22-Rki as an *Eco*RV/*Hind*III digested fragment and cloned into pBAD33 previously digested with the same enzymes.

To construct pBAD24-Rki^Flag^, containing Rki with the N-terminal Flag tag, the *rki* gene was first PCR-amplified from pBAD22-Rki using primers RkiFlag-For (5′-GCTCCATGGCAAGCGACTACAAAGATGACGACGATAAAAGCGGCATGATTAACGAAAAAATGACACAT) and Rki-Rev (5′- GAAAGCTTGGATCCTT ATGCGTCTAAGTGCTTGCGGAAAG). The resulting 749 bp fragment was digested with *Nco*I and *Bam*HI and ligated into pBAD24 previously digested with the same enzymes. The same procedure was applied to construct pBAD24-Rki(H38Q)^Flag^ using pBAD22-Rki(H38Q) as template. To construct pBAD33-Rki^Flag^, the *rki* ^Flag^ gene from pBAD24-Rki^Flag^ was subcloned as an *Eco*RV/*Hind*III digested fragment into pBAD33 previously digested with the same enzymes.

To obtain p29SEN-Rki, the *rki* gene was PCR-amplified using primers EPw57f (5′-CGCAATTGTCATGATTAACGAAAAAATGACA) and rkiCter-rev (5′- GCAAGCTTGGATCCTTATGCGTCTAAGTGCTTGCG) and pBAD22-Rki as template. The resulting 733 bp fragment was digested with *Mfe*I and *Hind*III and ligated into p29SEN digested with the same enzymes. The same procedure was followed for p29SEN-Rki(H38Q) with pBAD22-Rki(H38Q) as template.

Construction of pET15b-Rki^His6^ expressing Rki with an N-terminal 6×His tag was performed as follows. Both primers HisRB43Jfor (5′-GCCCCATGGGCAGCAGCCATCATCATCATCATCATAGCAGCATGATTAACGAAAAAATGACACAT-3′) and RB43J-rev (5′-GCAGATCTAAGCTTTATGCGTCTAAGTGCTTGCG) were used to PCR amplify *rki* from pBAD22-Rki. The resulting *rki-his* PCR fragment was cloned as an *Nco*I/*Hind*III fragment into pBAD24 previously digested with the same enzymes. The *rki-his* gene was then subcloned as an *Nco*I/*Bgl*II digested fragment into pET15b vector (Novagen) previously digested with the same enzymes. The same procedure was used to construct pET15b-Rki(H38Q)^His6^ , except that pBAD22-Rki(H38) was used as DNA template.

The Rki-DnaJ and Rki(H38Q)-DnaJ chimeras containing the 77 amino acid long N-terminal J-domain sequence of Rki grafted into *E. coli'*s DnaJ were constructed as described (Kelley and Georgopoulos, 1997). Briefly, the 231 bp long fragment containing the Rki J-domain was PCR amplified using primers BR43DnaJfor and SRB43JKpnIrev (5′-GCGGTACCCGCTGCGTGACGCGCTCGCAT), and RB43 DNA as template. The PCR products were cloned as *Eco*RI/*Kpn*I fragements into pWKG90KPN plasmid [30].

To construct p29SEN-RpoH, the 855 bp long *rpoH* gene was PCR-amplified using primers rpohfor (5′-GAGAATTCCATATGACTGACAAAATGCAAAG) and rpohrev (5′-GAAAGCTTGGATCCTTACGCTTCAATGGCAGCAC), and *E. coli* MG1655 genomic DNA as template. The PCR fragment was ligated as an *Eco*RI/*Hind*III fragment into *Eco*RI/*Hind*III p29SEN. All the constructs obtained by PCR were sequenced verified using the appropriate primers.

### *In vivo* assays

Bacterial motility and bacteriophage λ plating assays were performed at 30°C as described [29]. To monitor bacterial viability, mid-log phase cultures of fresh transformants grown in LB medium (tryptone 10 g/L, NaCl 10 g/L, yeast extract 5 g/L, thymine 20 g/L, NaOH 3 mM) were serially diluted and spotted on LB agar plates (agar 15 g/L) supplemented when necessary with the appropriate antibiotics (100 µg/ml ampicillin, 20 µg/ml chloramphenicol, 20 µg/ml kanamycin) and various L-arabinose or IPTG-inducers. Note that 0.2% glucose was added to overnight cultures in order to prevent Rki toxicity. RB43 plaque-forming ability was monitored as follows. Overnight cultures of *E. coli* grown in LB medium were diluted 1∶100 and grown with shaking to an OD_600_ of 0.7 at the indicated temperature. Cultures were then concentrated 10-folds in the same medium and bacterial lawns were prepared by mixing 300 µl of cells with 3 ml of pre-warmed H-Top medium (tryptone 10 g/L, NaCl 8 g/L, Na citrate 2.4 g/L, glucose 3 g/L, NaOH 0.5 mM, agar 7 g/L) and subsequently pouring the mix onto LB agar plates. Both, H-Top and LB plates were supplemented with the appropriate antibiotics and/or inducers when necessary. Serial dilutions of bacteriophage lysates were then spotted on bacterial lawns and plates incubated as indicated in the figure legends.

### Immunoprecipitation

Cells were grown in 100 ml LB supplemented with the appropriate antibiotics to an OD_600_ of 1.2 and harvested by centrifugation at 7000 rpm for 30 min at 4°C in a Beckman JA14 rotor. Pellets were resuspended in 1 ml of IP buffer (50 mM Tris-HCl buffer, pH 7.5, 0.15 M NaCl, 20% (v/v) glycerol, 10 mM PMSF, 1 µl/ml Benzonase, 1 mg/ml lysozyme), sonicated twice for 20 sec and centrifuged at 14000 *g* for 30 min at 4°C. Supernatants were incubated 30 min at 25°C and 10 mM ADP, Hexokinase and 0.2% glucose was added for 15 min at 20°C. The samples were then incubated at 4°C for 2 h with 25 µl of anti-Flag M2-agarose suspension (Sigma), washed with 6 ml TBS (50 mM Tris-HCl buffer, pH 7.5 and 0.15 M NaCl), and the bound proteins were eluted with 30 µl of TBS containing 5 µg of FLAG peptide (Sigma). Proteins were separated by sodium dodecyl sulfate-polyacrylamide gel electrophoresis (SDS-PAGE) (4–12% Biorad).

### Purification of RNA from RB43-infected cells

Bacterial cultures (16 ml/infection) were grown at 30°C in LB medium to a density of 4.10^8^ cells/ml. Cells were concentrated 4-fold in the same medium and bacteriophage infections were initiated by mixing 4 ml of cells with the adequate volume of RB43 bacteriophage stock (multiplicity of infection (MOI) of 10). Cells were then incubated at 30°C with shaking and infections were stopped at the desired time by adding 400 µl of pre-heated RNA lysis buffer (0.5 M Tris-HCl, 20 mM EDTA, 10% SDS, pH 6.8) and incubating in boiling water for 2 min. One volume of phenol was added and after mixing one volume of chloroform. Samples were incubated for 10 min at 30°c under shacking and centrifuged in order to collect the aqueous phase. Three more phenol/chloroform extractions were carried out. Nucleic acids were precipitated by mixing 2.5 volumes of prechilled ethanol (−20°C) in the presence of 0.3 M sodium-acetate at pH 5.7 and incubating at −20°C for at least 1 h. The pellet was collected by centrifugation (8000 rpm, 30 min), dried and resuspended in 250 µl of DEPC treated water. Small samples (usually 5 µl) were examined for RNA degradation and DNA contamination by electrophoresis on agarose gel containing ethidium bromide. Each infection allowed us to collect 200 to 300 µg of viral RNA.

### RNA electrophoresis and Northern blotting

50 µg of RB43 RNA, prepared as described above were treated with 100 units of DNAse I FPLCpure (Amersham) and precipitated by mixing 2.5 volumes of prechilled (−20°C) ethanol and 0.3 M sodium-acetate at pH 5.7 and incubating at −20°C for at least 1 h. Pellet was collected by centrifugation (8000 rpm, 30 min), dried and resuspended in 17.5 µl of DEPC treated water. A mix containing 10 µl of 10× MOPS, 17.5 µl of formaldehyde and 50 µl of formamide was prepared and mixed to the resuspended RNA. Samples were then incubated at 55°C for 15 min. Ten µl of 10× RNA loading buffer (1 mM EDTA pH 8, 50% (v/v) glycerol, 0.25% bromophenol blue , 0.25% xylene cyanol FF) and 5 µl of ethidium bromide (10 mg/ml) were added to the mix. 10 µl of each samples were used for adjusting the loading amount of RNA by running on an agarose gel. Samples (usually 15 µl) were then electrophoresed at room temperature for 3 h in 1% agarose gel (180 ml final volume) prepared in 1× MOPS containing 9.7 ml of formaldehyde. RNA integrity was verified under UV lamps. The resolved RNA population was subsequently transferred on a positively charged nylon membrane (Hybond-N^+^ membrane from Amersham pharmacia biotech) by salt diffusion over-night. RNA was UV cross-linked to the membrane and the efficiency of transfer was examined by methylene blue staining. The membrane was then prehybridized for 30 min at 68°C in hybridization buffer (7% SDS, 250 mM NaPi (0.77 M Na_2_HPO_4_/0.22 M NaN_2_PO_4_ mix), 2 mM EDTA). Prehybridization buffer was discarded and replaced by fresh buffer. ^32^P-labeled probe (see below) was added and hybridization was carried out over night at 68°C. The membrane was then washed twice at 60°C in a 5% SDS, 250 mM NaPi and 2 mM EDTA solution for 20 min and once in a 1% SDS, 250 mM NaPi, 2 mM EDTA solution for 30 min. Detection of the signals on autoradiograms was performed by exposure of the membrane at −80°C for 5–12 h in presence of an intensifying screen.

### Probe labeling

Probes consisted of PCR products obtained by amplifying the desired fragments from the RB43 genomic DNA using the pfu polymerase. The PCR products were purified from 1.5% agarose gels using the Qiaquick gel extraction kit from Qiagen (cat. No.28706). 2 µl of the PCR products were used as a template for a new PCR reaction containing ^32^P-αdCTP (1.5 mM MgCl_2_, 2 µM of each primers, 2 mM of dATP, dTTP, dGTP, 0.2 mM dCTP (0.1 mM), 5 µl of ^32^P-αdCTP (10 µCi/µl), 5 units of Hot-start Taq (Qiagen,1× buffer). The PCR products were separated from free radioactivity by using Qiaquick gel extraction kit according to the furnisher. 100 µl of sonicated salmon sperm DNA (10 mg/ml) were added and the mixture was incubated at 95°C for 2 min and then diluted in 1 ml of hybridization buffer. Location of the different primers used is indicated in Figure 4 and their respective sequences were: for gene *43* probe (700 bp long): 43gp43.0 (5′-ATGAATGAATTTTATCTATCA-3′) and 43gp43.3 (5′-CACGCCATAAATTTCGTATCC-3′). For gene *37* probe (342 bp long): 43gp372am6 (5′-TAATTTGCCTTTACTCCCTACTGGA-3′) and 43gp372am3 (5′- GGATCGGAAGTATTCTATTTTGTGTT-3′). For *ORF057* probe (480 bp long) : RB43Jfor (5′-GCGAATTCATGATTAACGAAAAAATGACA-3′) and 43gpJDTM2 (5′- CCAAGCTTACATCAAACCTTTACCTTCTTC-3′).

### Protein purification

To avoid the toxic effect of protein overexpression in wild type *E. coli*, Rki and RkiH38Q were purified from the BL21Δ*dnaKdnaJ* strain [33]. Fresh overnight cultures were diluted 1∶100 in 500 ml of LB broth supplemented with 100 µg/µl ampicillin and grown with vigorous shaking at 30°C. At an OD_600_ of 0.3, 2 mM IPTG was added for 2 h. Cells were harvested at 7000 rpm for 30 min at 4°C in a Beckman JA14 rotor and pellets were stored at −80°C. Pellets were resuspended in 20 ml of lysis buffer (50 mM NaH2PO4, pH 8.0, 300 mM NaCl, 10 mM Imidazole), 1 mg/ml lysozyme was then added and the cell suspensions were kept on ice for 30 min. After addition of Protease Inhibitor (Roche), cells were sonicated 6×20 s on ice and centrifuged at 12000 rpm for 30 min at 4°C in a Beckman JA25.50 rotor. Supernatants were collected, 45% ammonium sulfate was added and samples were incubated overnight at 4°C under mild shaking. Samples were then centrifuged at 10000 rpm for 10 min at 4°C in Beckman JA25.50 rotor. The supernatants were dialyzed twice for 2 h in 2 L of lysis buffer in a Spectra/Por® Membrane, MWCO 12–14000 cut off 3.5 kDa. The dialysates were applied to a 4 ml of nickel-nitrilotriacetic acid columns preequilibrated with 10 ml of lysis buffer. The following steps were performed as described in the procedure from Qiagen for the purification of His6-tagged proteins from *E. coli* using nickelnitrilotriacetic acid superflow under native conditions, using lysis buffer supplemented with 20 mM imidazole as washing buffer and using 250 mM imidazole as elution buffer. The proteins were stored at −80°C in buffer containing 25 mM HEPES buffer, pH 7.6, 0.4 M KCl, 1 mM DTT, 10% (v/v) glycerol. DnaK purification was performed as described [33], and DnaJ and GrpE were purchased from Stressgen.

### ATPase activity

ATPase activity was essentially carried out as described [52], with minor modifications. Reactions were performed in 10 µl reaction buffer (30 mM HEPES buffer, pH7.6, 40 mM KCl, 10 mM NaCl, 4 mM MgAc, 2 mM DTT, 0.29 mg/ml BSA, 0.1 mM ATP) in presence of 1 µM DnaK, 1 µM GrpE, 1 µCi [γ^32^P]ATP, and increasing concentrations (0.2, 0.4, 0.6 or 0.8 µM) of DnaJ, Rki or RkiH38Q. Three µl of 0 or the 20 min reaction were spotted on thin layer chromatography and migrated in migration buffer containing 0.15 M LiCl and 0.15 M formic acid. The amount of liberated γ-phosphate was quantified using phosphrimaging.

### Luciferase aggregation and refolding assays

Firefly luciferase aggregation was performed as described [33], except that luciferase was denatured for 90 min at 25°C and aggregation kinetics were followed at 25°C. The protein concentrations used are described in the figure legend. Reactivation of firefly luciferase was performed essentially as described [33]. Briefly, 25 µM luciferase (Sigma) was denatured for 2 h at 22°C in 30 mM Tris-HCl buffer, pH 7.6, 6 M guanidinium chloride, 5 mM DTT. Denatured luciferase was diluted to a final concentration of 0.125 µM into a reaction mixture (50 µl final) containing 100 mM MOPS, 500 mM KCl, 50 mM MgCl_2_, 20 mM creatine phosphate, 0.1 mg.mL^−1^ creatine kinase, 5 mM ATP, 0.015% bovine serum albumin, 0.5 µM DnaK and 0.125 µM GrpE. All components were incubated on ice. Refolding was initiated by adding either DnaJ or DnaJ mutant protein (0.125 µM each). The luciferase activity was measured at different time points after incubation at 22°C by using 10 µL of the luciferase assay system from Promega (E1500) and a Berthold Centro LB960 luminometer.

## Supporting Information

Figure S1 DnaK-dependent toxicity of Rki and the role of its C-terminal domain. (A) Rki and Rki(H38Q) expression in wild-type and Δ*dnaK* strains. An immunoblot analysis of whole cell extracts showing the steady-state expression levels of Rki and Rki(H38Q) is presented. The arabinose inducible pBAD22-based full-length Rki constructs were expressed at 30°C in wild-type or in the Δ*dnaK*52::Cm^R^ isogenic strain in the presence of 1% of L-arabinose inducer as described in Figure 2B. A control immunoblot showing the presence or the absence of endogenous DnaK is also presented. (B) Complementation of the temperature-sensitive phenotype of the *E. coli* triple J-domain mutant strain W3100 Δ3 (Δ*cbpA* Δ*djlA dnaJ*::Tn*10*-42) by pBAD22-based JDP constructs, DnaJ, Rki full-length and Rki(1–159) truncated for its last 78 amino acids. Mid-log phase cultures of the transformants were serial diluted and spotted on LB amp plates without or with L-arabinose inducer at the indicated concentration. Plates were incubated overnight at 30° and 43°C. (C) Coomassie-stained SDS-PAGE showing the steady state levels of the pBAD22-based DnaJ chimeras expressed in strain W3100 Δ3 at 30°C in the presence of 0.5% L-arabinose.
(TIF)

Figure S2 Biochemical analyses of the Rki protein. (A) A SEC-MALLS experiment showing the light scattering signal (red trace) and the differential refractive index signal (green trace) against elution time (min) for Rki. The blue trace represents the molar mass (g.mol^−1^) calculated across the elution peak according to SLS measurement whereas the average molar mass (3.15×10^4^ g.mol^−1^) is indicated by a dashed black line. (B) Partial α-Chymotrypsin proteolysis followed by N-terminal Edman sequencing of purified his-tagged Rki wild-type protein. Samples were subjected to 12% SDS-PAGE and subsequently stained with Coomassie blue. The amino acid sequences obtained after N-terminal Edman sequencing of the digested fragments are shown on the right panel.
(TIF)

Figure S3 Bacteriophage T4 and RB43 growth at various temperatures. Ten-fold serial dilutions of T4 and RB43 stocks were spot tested on bacterial lawns of *E. coli* strain W3110 and incubated overnight at 37° and 43°C or for 2 days at 16°C.
(TIF)

Figure S4 Complementation assay for bacteriophage RB43Δ*rki* plaque formation on the W3110 strain. (A) RB43Δ*rki* plaque-forming ability was monitored as described above on W3110 transformed either with the plasmid pBAD22 empty vector, or pBAD22-Rki or pBAD-Rki(H38Q) in the presence of 0.5% L-arabinose inducer and incubated for 2 days at 14°C. (B) An immunoblot analysis of whole cell extracts from (A) showing the steady-state expression levels of Rki and Rki(H38Q).
(TIF)

Figure S5 Co-expression of σ^32^ aggravates Rki toxicity in *E. coli*. (A) Mid-log phase cultures of W3110 co-transformed with p29SEN-RpoH (σ^32^) and pBAD33 or pBAD33-Rki were serial diluted and spotted on LB amp cm plates supplemented with 0.5 mM IPTG to induce σ^32^ expression, with or without L-arabinose inducer at the indicated concentration. Plates were incubated overnight at 30°C. (B) Control immunoblot analysis showing the steady state expression level of Rki with (Rki + σ^32^) or without (Rki) co-expressed σ^32^ in the presence of 0.5 mM IPTG and 0.1% L-arabinose inducers at 30°C.
(TIF)

Figure S6 (A) Immunoblot analysis showing DnaK endogenous level in FA1195 P_tet_ *dnaKJ* whole cell extracts at 30°C in the absence of anhydrotetracycline under the growth conditions used in Figure 6E. The wild-type strain grown under the same conditions is shown as a positive control. (B) Suppression of the bacteriophage RB43Δ*rki* growth phenotype by GroEL/GroES overproduction. RB43Δ*rki* plaque-forming ability was monitored on strain MC4100 transformed with either the plasmid p29SEN empty vector or p29SEN-GroESL in the presence of 100 µM IPTG inducer.
(TIF)

Figure S7 Co-expression of ORF58 does not affect Rki function. (A) Cell lysates collected after 30 min of infection of W3310 by either bacteriophage RB43, RB42 or RB16 were separated by SDS-PAGE. Rki and Rki-58 fusion (Rki16) proteins were revealed by western blot analysis using anti-Rki antibody. (B) Western blot analysis (probed with anti-Rki or anti-σ^32^ antibodies) of whole cell extracts from strain MC4100 co-transformed with pMPMK6-ORF58 and either p29SEN empty vector, p29SEN-Rki or p29SEN-Rki16, and grown for 1 h with the indicated inducers concentration (50 µM IPTG, 0.5% L-ara). (C) Complementation assay for RB43Δ*rki* plaque formation on MC4100 strain transformed with plasmids p29SEN empty vector, p29SEN-Rki, p29SEN-Rki(H38Q) or p29SEN-Rki16 without or with 50 µM of IPTG inducer. ND stands for not determined (due to Rki toxicity). (D) LMG190 strain transformed with the same set of plasmids as in (B) were grown overnight, serially diluted ten-fold and spotted on LB amp kan agar plates supplemented with 0.5% L-arabinose and the indicated µM concentration of IPTG (µM). (E) Whole cell extract of the same transformants as in (B) were separated by SDS-PAGE and stained with Coomassie blue.
(TIF)

Text S1 Supplementary Materials and Methods.
(DOCX)
