# Supplementary figures and images for: A Bacteriophage-Encoded J-Domain Protein Interacts with the DnaK/Hsp70 Chaperone and Stabilizes the Heat-Shock Factor σ32 of Escherichia coli
Source: PLoS Genet. 2012 Nov 1;8(11):e1003037. doi: 10.1371/journal.pgen.1003037 (PMC3486835; doi:10.1371/journal.pgen.1003037)

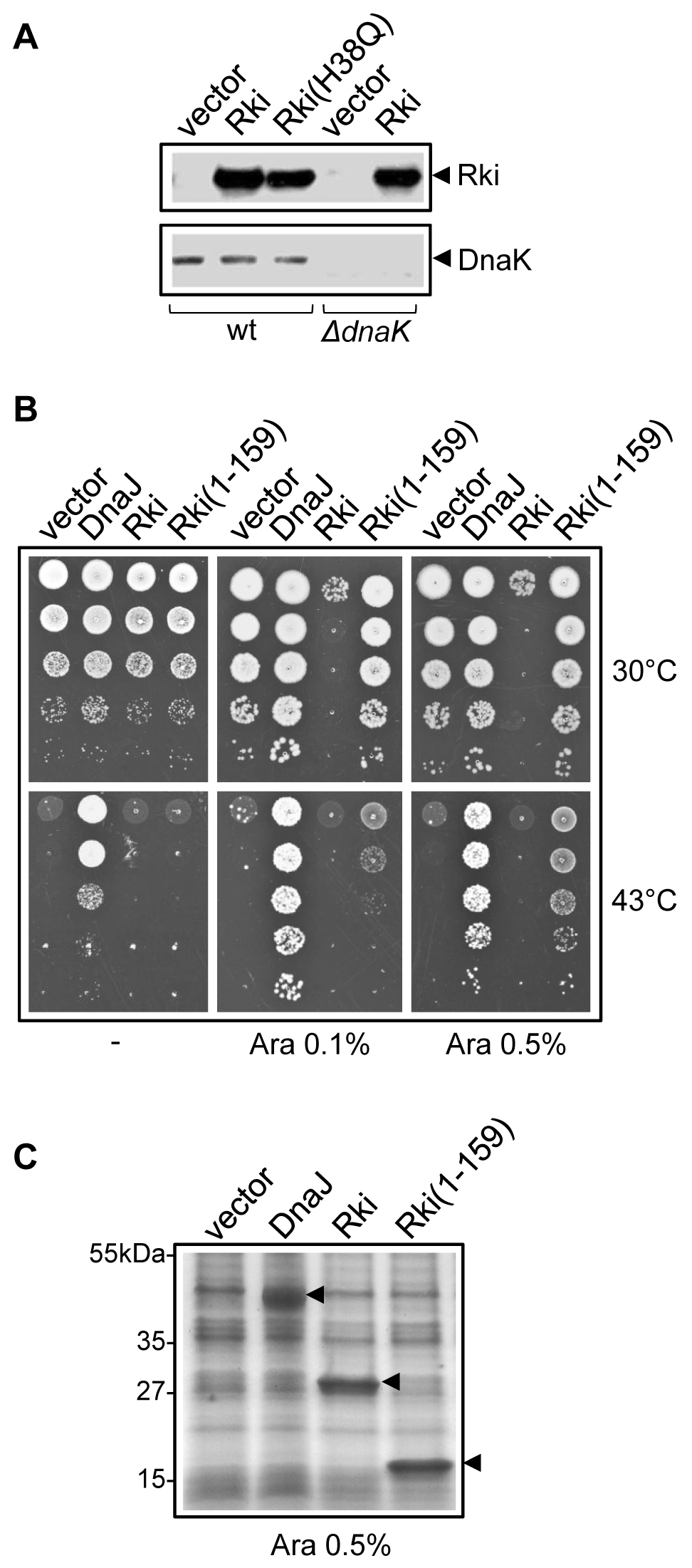

Supplement: Figure S1 — DnaK-dependent toxicity of Rki and the role of its C-terminal domain. (A) Rki and Rki(H38Q) expression in wild-type and ΔdnaK strains. An immunoblot analysis of whole cell extracts showing the steady-state expression levels of Rki and Rki(H38Q) is presented. The arabinose inducible pBAD22-based full-length Rki constructs were expressed at 30°C in wild-type or in the ΔdnaK52::CmR isogenic strain in the presence of 1% of L-arabinose inducer as described in Figure 2B. A control immunoblot showing the presence or the absence of endogenous DnaK is also presented. (B) Complementation of the temperature-sensitive phenotype of the E. coli triple J-domain mutant strain W3100 Δ3 (ΔcbpA ΔdjlA dnaJ::Tn10-42) by pBAD22-based JDP constructs, DnaJ, Rki full-length and Rki(1–159) truncated for its last 78 amino acids. Mid-log phase cultures of the transformants were serial diluted and spotted on LB amp plates without or with L-arabinose inducer at the indicated concentration. Plates were incubated overnight at 30° and 43°C. (C) Coomassie-stained SDS-PAGE showing the steady state levels of the pBAD22-based DnaJ chimeras expressed in strain W3100 Δ3 at 30°C in the presence of 0.5% L-arabinose. (TIF) [file pgen.1003037.s001.tif]

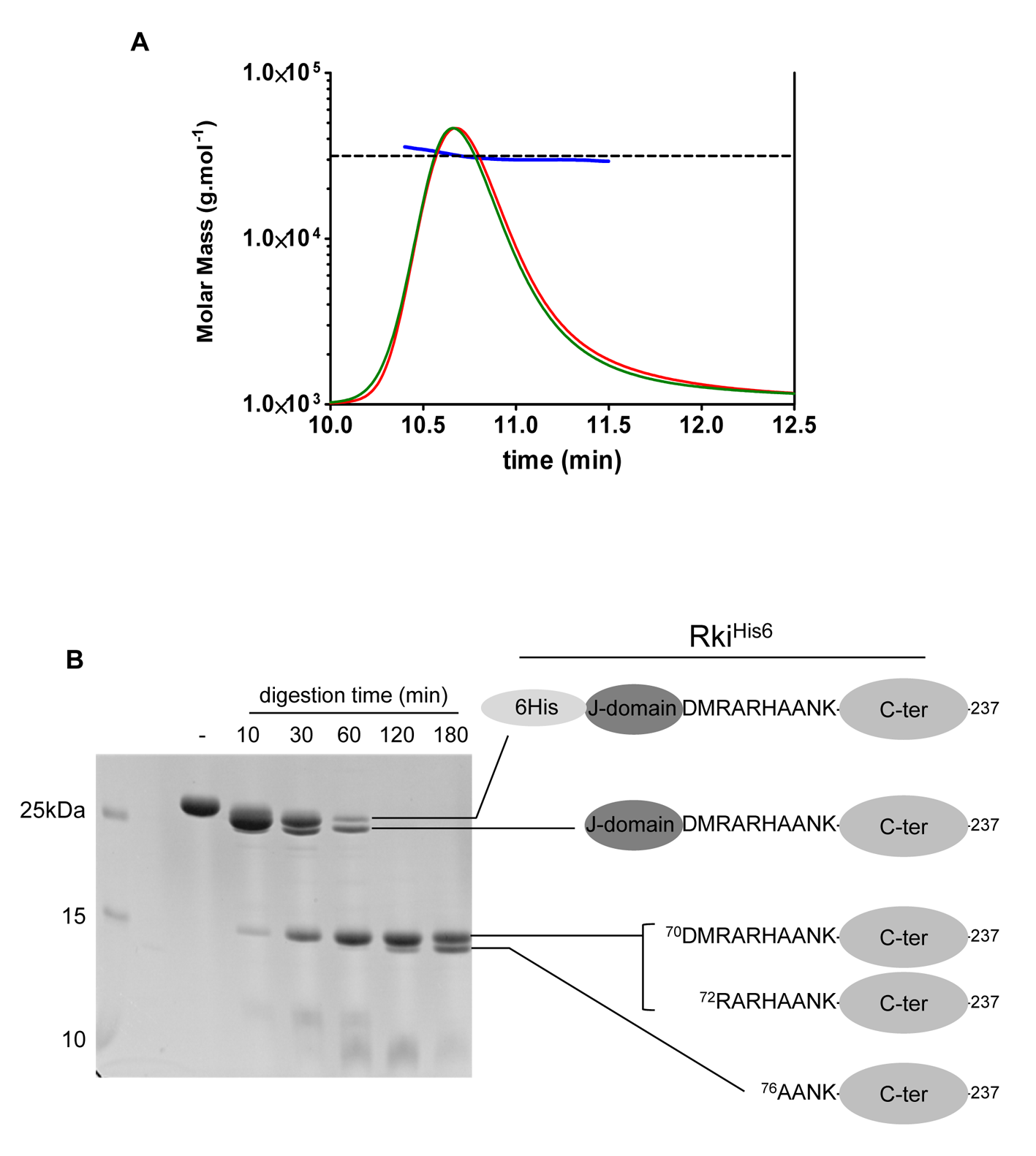

Supplement: Figure S2 — Biochemical analyses of the Rki protein. (A) A SEC-MALLS experiment showing the light scattering signal (red trace) and the differential refractive index signal (green trace) against elution time (min) for Rki. The blue trace represents the molar mass (g.mol−1) calculated across the elution peak according to SLS measurement whereas the average molar mass (3.15×104 g.mol−1) is indicated by a dashed black line. (B) Partial α-Chymotrypsin proteolysis followed by N-terminal Edman sequencing of purified his-tagged Rki wild-type protein. Samples were subjected to 12% SDS-PAGE and subsequently stained with Coomassie blue. The amino acid sequences obtained after N-terminal Edman sequencing of the digested fragments are shown on the right panel. (TIF) [file pgen.1003037.s002.tif]

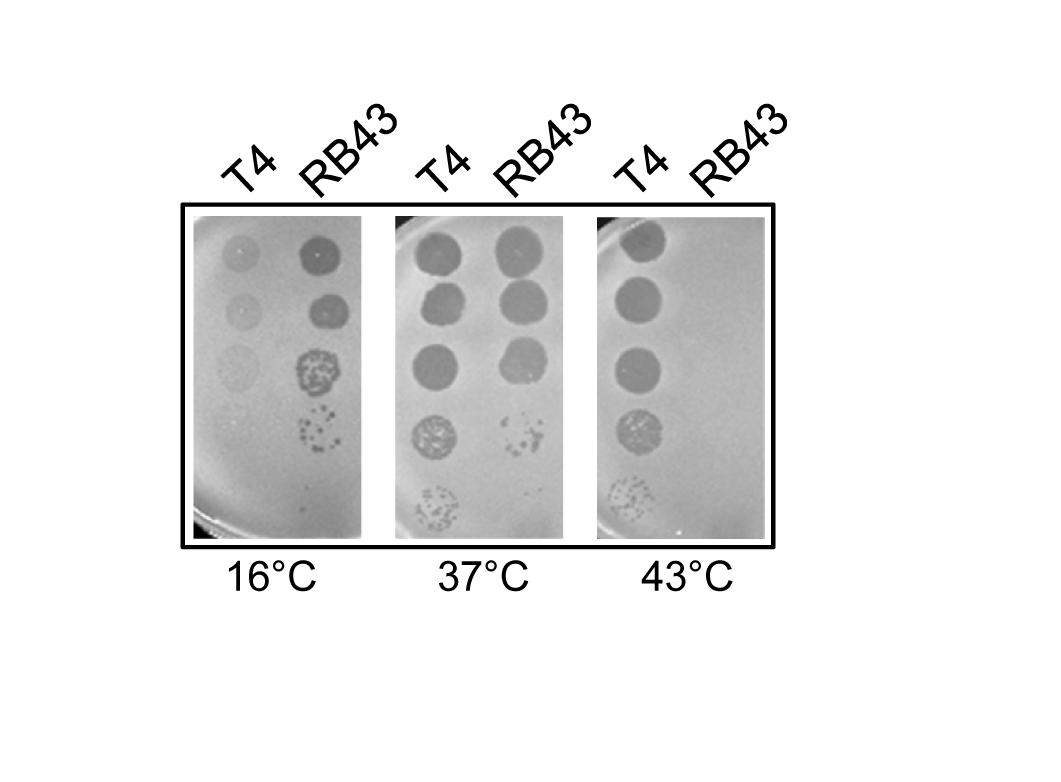

Supplement: Figure S3 — Bacteriophage T4 and RB43 growth at various temperatures. Ten-fold serial dilutions of T4 and RB43 stocks were spot tested on bacterial lawns of E. coli strain W3110 and incubated overnight at 37° and 43°C or for 2 days at 16°C. (TIF) [file pgen.1003037.s003.tif]

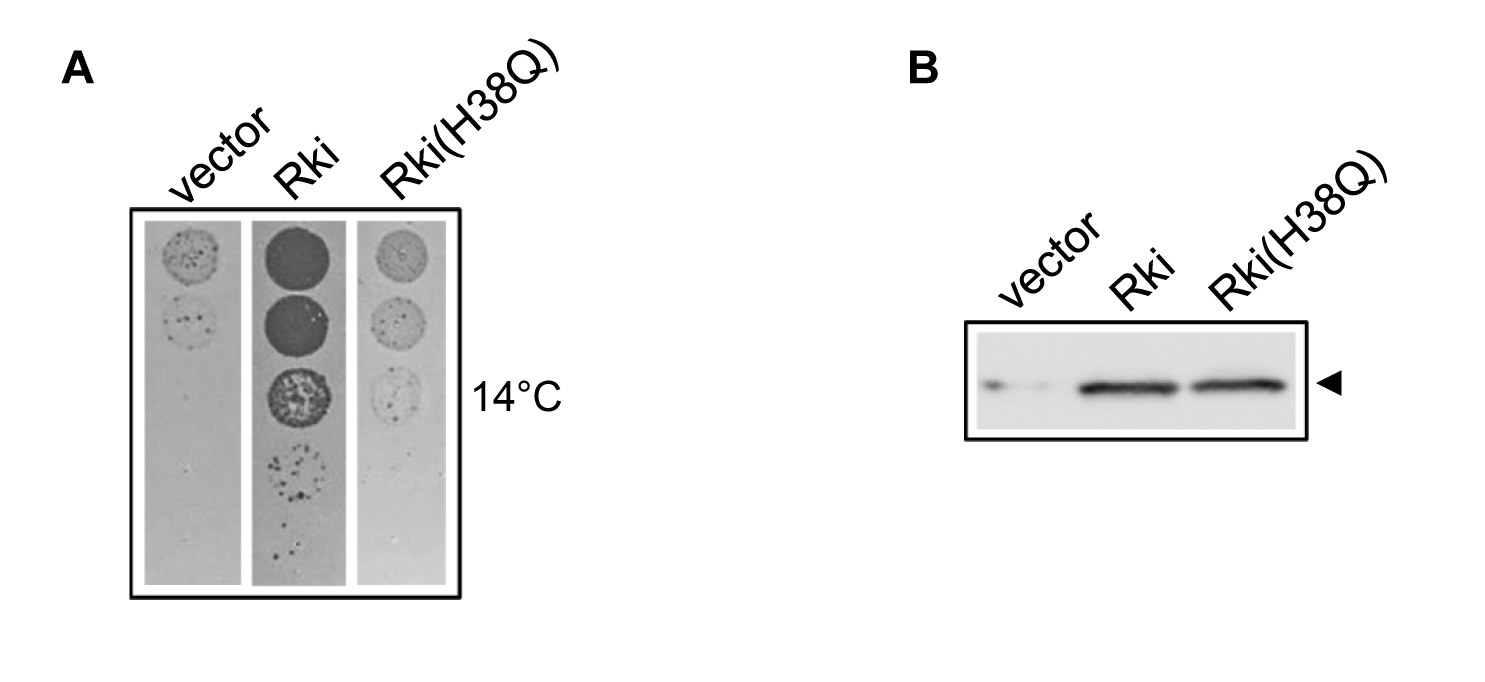

Supplement: Figure S4 — Complementation assay for bacteriophage RB43Δrki plaque formation on the W3110 strain. (A) RB43Δrki plaque-forming ability was monitored as described above on W3110 transformed either with the plasmid pBAD22 empty vector, or pBAD22-Rki or pBAD-Rki(H38Q) in the presence of 0.5% L-arabinose inducer and incubated for 2 days at 14°C. (B) An immunoblot analysis of whole cell extracts from (A) showing the steady-state expression levels of Rki and Rki(H38Q). (TIF) [file pgen.1003037.s004.tif]

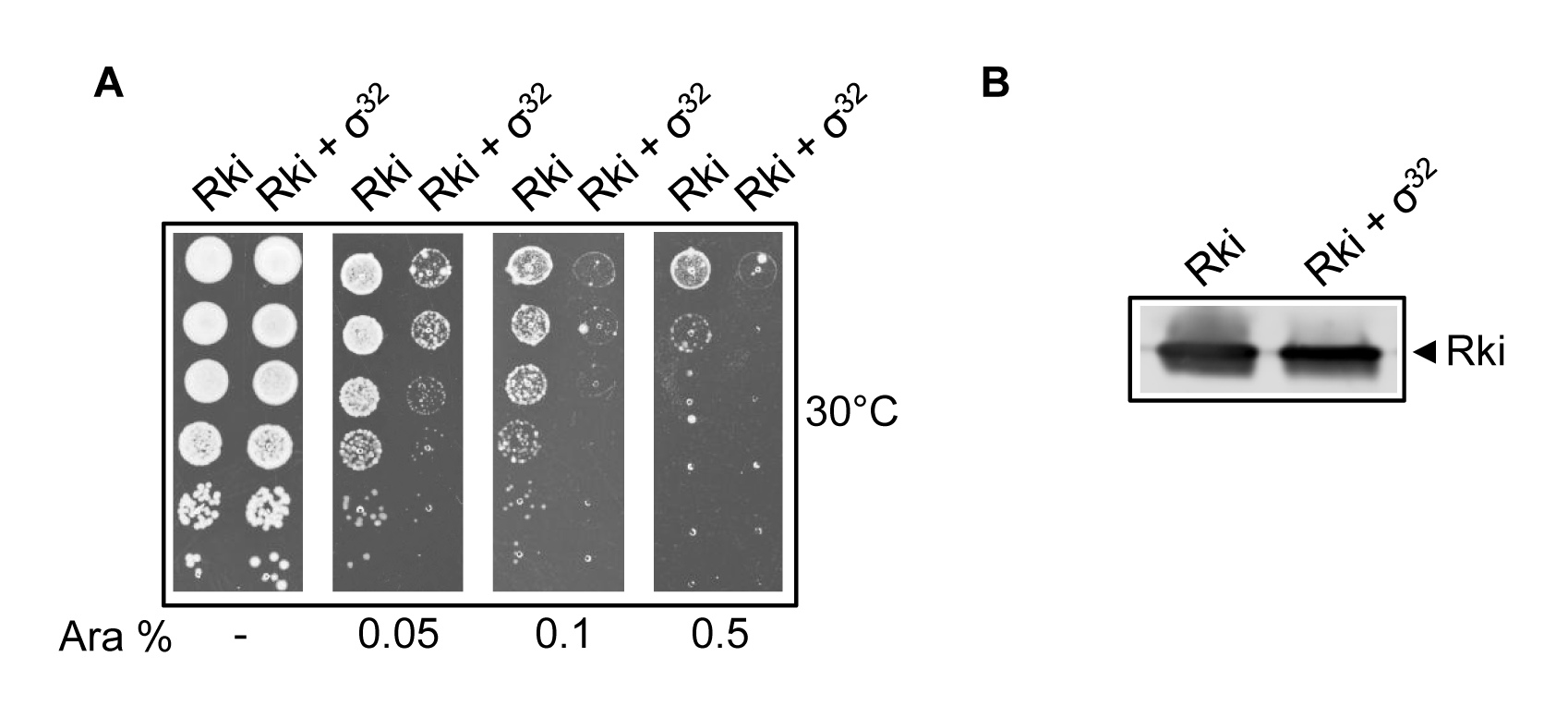

Supplement: Figure S5 — Co-expression of σ32 aggravates Rki toxicity in E. coli. (A) Mid-log phase cultures of W3110 co-transformed with p29SEN-RpoH (σ32) and pBAD33 or pBAD33-Rki were serial diluted and spotted on LB amp cm plates supplemented with 0.5 mM IPTG to induce σ32 expression, with or without L-arabinose inducer at the indicated concentration. Plates were incubated overnight at 30°C. (B) Control immunoblot analysis showing the steady state expression level of Rki with (Rki + σ32) or without (Rki) co-expressed σ32 in the presence of 0.5 mM IPTG and 0.1% L-arabinose inducers at 30°C. (TIF) [file pgen.1003037.s005.tif]

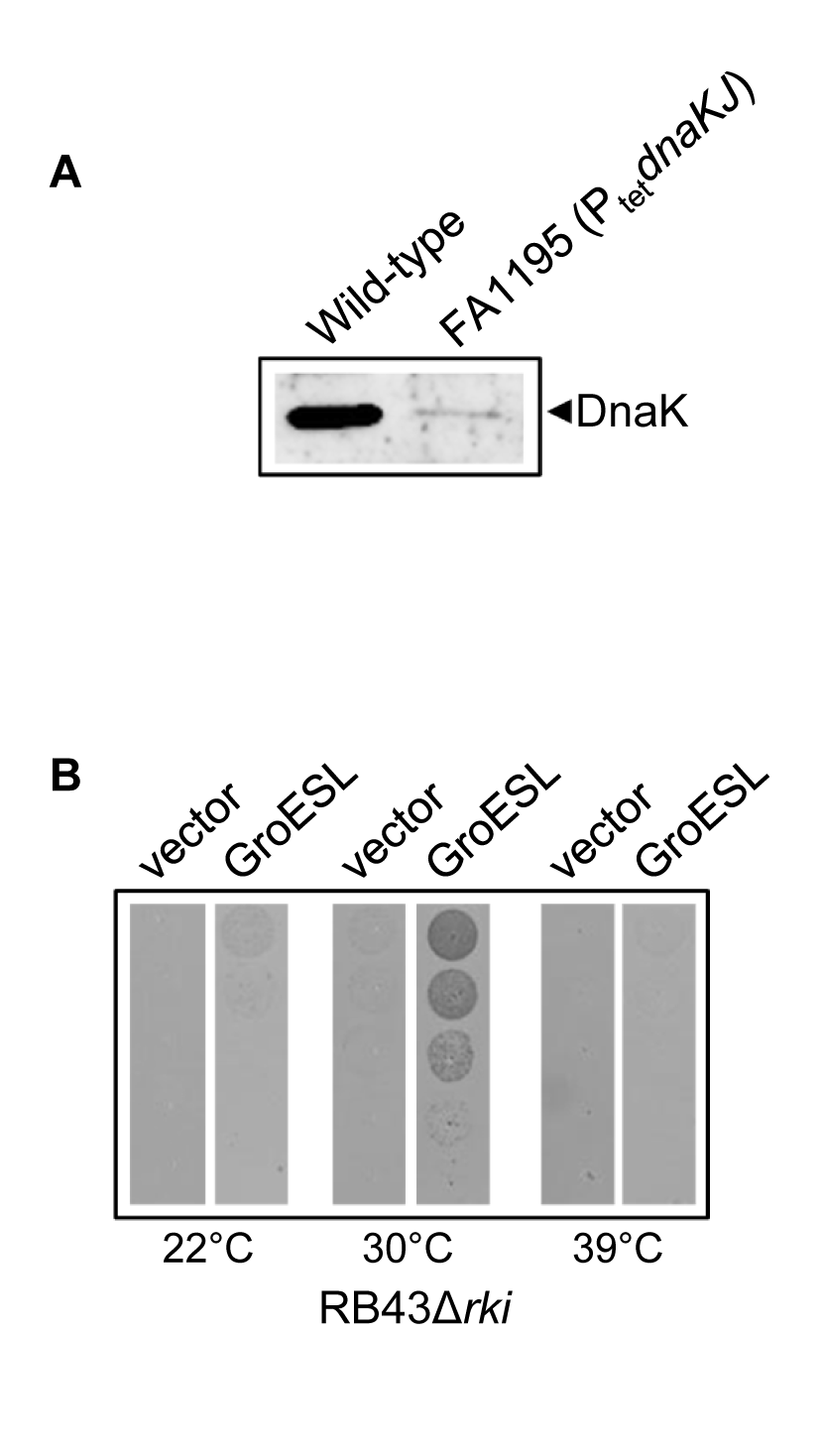

Supplement: Figure S6 — (A) Immunoblot analysis showing DnaK endogenous level in FA1195 Ptet dnaKJ whole cell extracts at 30°C in the absence of anhydrotetracycline under the growth conditions used in Figure 6E. The wild-type strain grown under the same conditions is shown as a positive control. (B) Suppression of the bacteriophage RB43Δrki growth phenotype by GroEL/GroES overproduction. RB43Δrki plaque-forming ability was monitored on strain MC4100 transformed with either the plasmid p29SEN empty vector or p29SEN-GroESL in the presence of 100 µM IPTG inducer. (TIF) [file pgen.1003037.s006.tif]

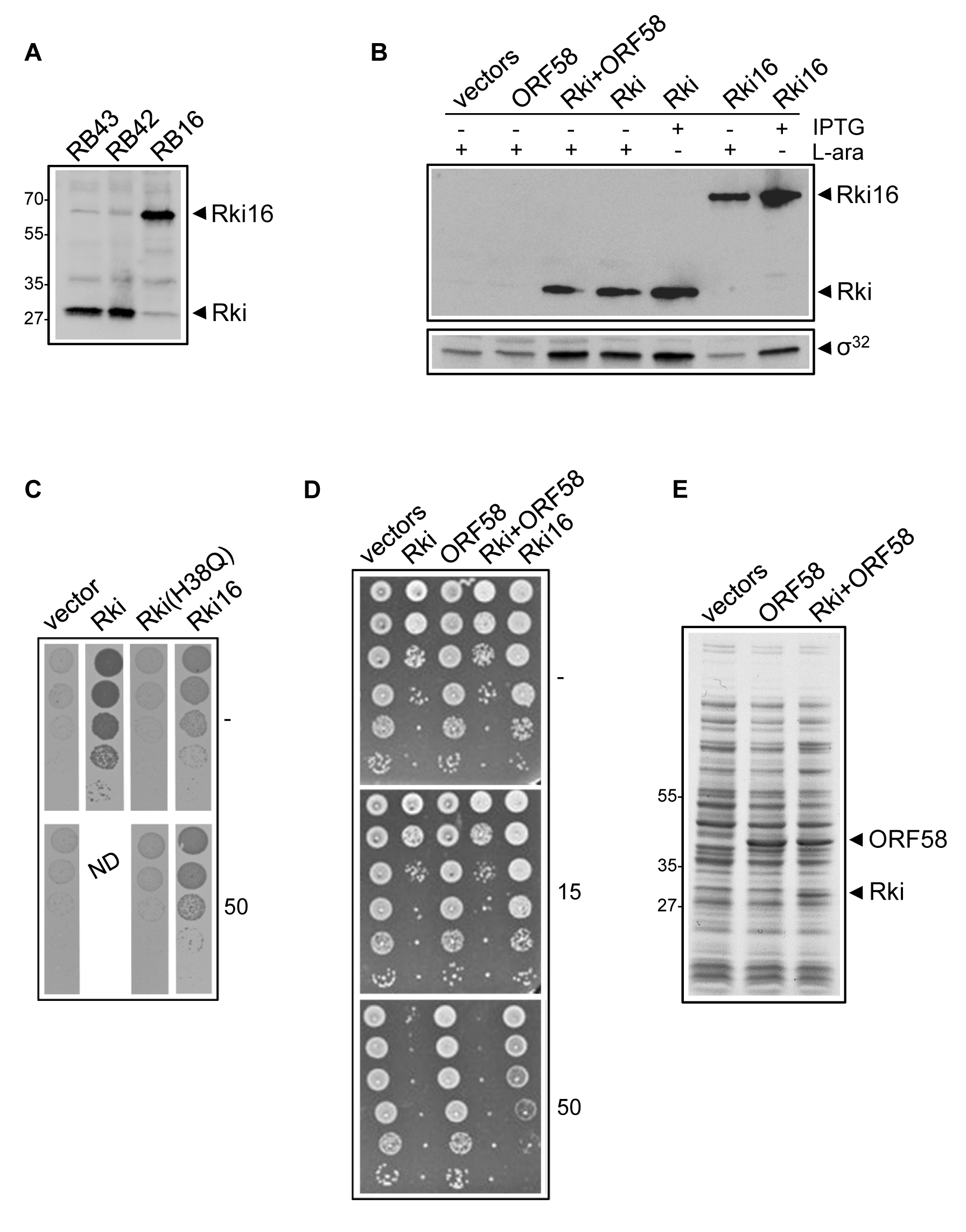

Supplement: Figure S7 — Co-expression of ORF58 does not affect Rki function. (A) Cell lysates collected after 30 min of infection of W3310 by either bacteriophage RB43, RB42 or RB16 were separated by SDS-PAGE. Rki and Rki-58 fusion (Rki16) proteins were revealed by western blot analysis using anti-Rki antibody. (B) Western blot analysis (probed with anti-Rki or anti-σ32 antibodies) of whole cell extracts from strain MC4100 co-transformed with pMPMK6-ORF58 and either p29SEN empty vector, p29SEN-Rki or p29SEN-Rki16, and grown for 1 h with the indicated inducers concentration (50 µM IPTG, 0.5% L-ara). (C) Complementation assay for RB43Δrki plaque formation on MC4100 strain transformed with plasmids p29SEN empty vector, p29SEN-Rki, p29SEN-Rki(H38Q) or p29SEN-Rki16 without or with 50 µM of IPTG inducer. ND stands for not determined (due to Rki toxicity). (D) LMG190 strain transformed with the same set of plasmids as in (B) were grown overnight, serially diluted ten-fold and spotted on LB amp kan agar plates supplemented with 0.5% L-arabinose and the indicated µM concentration of IPTG (µM). (E) Whole cell extract of the same transformants as in (B) were separated by SDS-PAGE and stained with Coomassie blue. (TIF) [file pgen.1003037.s007.tif]
