# Supplementary material for: A Bacteriophage-Encoded J-Domain Protein Interacts with the DnaK/Hsp70 Chaperone and Stabilizes the Heat-Shock Factor σ32 of Escherichia coli
Source: PLoS Genet. 2012 Nov 1;8(11):e1003037. doi: 10.1371/journal.pgen.1003037 (PMC3486835; doi:10.1371/journal.pgen.1003037)
Supplement: Text S1 — Supplementary Materials and Methods. (DOCX) [file pgen.1003037.s008.docx]

**Text S1-Supplementary Materials and Methods**

**A bacteriophage-encoded J-domain protein interacts with the DnaK/Hsp70 chaperone and stabilizes the heat-shock factor σ^32^ of *Escherichia coli***

Elsa Perrody, Anne-Marie Cirinesi, Carine Desplats, France Keppel, Françoise Schwager, Samuel Tranier, Costa Georgopoulos and Pierre Genevaux

**Materials and Methods**

Strains and plasmids

*E. coli* LMG190 is an arabinose resistant derivative of MC4100 [[1](#_ENREF_1)]. NapIV was used as the host strain for bacteriophage RB16 [[2](#_ENREF_2)]. Plasmids p29SEN-GroESL [[3](#_ENREF_3)] and pMPMK5 [[4](#_ENREF_4)] have been described. Bacteriophages RB16 and RB42 were kind gifts of Jim Karam (Tulane University) and Henry Krisch (CNRS-Toulouse). To construct pBAD22-Rki(1-159) with the C-terminal end deletion, the 477 bp long *rki* fragment was PCR amplified using primers RB43DnaJfor (5’-GCGAATTCATGATTAACGAAAAAATGACA-3’) and RB43DnaJC2-rev(5’-CCAAGTTTACATCAAACCTTTACCTTCTTC-3’), digested with *Bsp*HI-*Hind*III and ligated into *Nco*I-*Hind*III digested pWKG90. To construct the p29SEN-Rki16 plasmid, the 1698 bp long ORF*058* (Uniprot D9ICB9) was PCR amplified using primers EPw57f and EPw58r (5’- GCAAGCTTGGATCCTTAGTGTTTCAAGCGCCAGAGAGC-3’) and a RB16 bacteriophage lysate as DNA template. The resulting PCR fragment was digested with *Mfe*I and *Hind*III and ligated into p29SEN previously digested with *Eco*RI and *Hind*III. To construct plasmid pMPMK6-ORF58, the 918 bp long ORF58 (Uniprot Q56BZ0) was PCR amplified from the RB43 bacteriophage genome using primers EPw58f (5’-CGCAATTGATATGCGTAAAGGTGCGTATGTG-3’) and EPw58r, digested with *Mfe*I and *Hind*III, and ligated into *Eco*RI/*Hind*III digested pMPMK6.

Size exclusion chromatography and multiangle laser light scattering (SEC-MALLS)

A Rki protein sample buffered in 25 mM Hepes buffer, pH 8.0, 150 mM KCl, 1 mM DTT was loaded on a BioSec-3 (300A) column (Agilent Technology, Massy, France) using an Agilent 1260 Infinity LC chromatographic system (Agilent Technology) coupled to a multiangle laser light scattering detection system. The column was equilibrated with a 0.1 µm filtered 50 mM Hepes buffer, pH 7.5, 150 mM NaCl. Data were collected using a DAWN HELEOS-8 (8 angles) and Optilab T-rEX refractive index detector (Wyatt Technology Corp., Toulouse France). Sample concentration was 2.5 g.l^-1^ for Rki solution. The protein sample was diluted in the Hepes buffer used in the mobile phase. Twenty µl of protein sample was loaded onto the column and the separation was performed at a flow rate of 0.3 ml.min^-1^ at 10°C. The results were analyzed using the ASTRA 6.0.2.9 software (Wyatt Technology Corp.).

α-Chymotrypsin proteolysis

Purified Rki_His6_ was incubated with α-Chymotrypsin (Sigma) (w/w 100/1) in reaction buffer (125 mM Hepes buffer pH 8.0, 0.2 M KCl, 5%(v%v) glycerol, 0.5 mM DTT) for 10, 30, 60, 120 or 180 min at 12°C. The reaction was stopped with 2 mM PMSF. Samples were migrated on a 12% SDS-PAGE and stained with Coomassie blue. For visualization of the fragments, the same reaction samples were transferred after migration on a PVDF membrane, stained with amido black and then subjected to Edman sequencing.

**References**

1. Guzman LM, Belin D, Carson MJ, Beckwith J (1995) Tight regulation, modulation, and high-level expression by vectors containing the arabinose PBAD promoter. J Bacteriol 177: 4121-4130.

2. Petrov VM, Nolan JM, Bertrand C, Levy D, Desplats C, et al. (2006) Plasticity of the gene functions for DNA replication in the T4-like phages. J Mol Biol 361: 46-68.

3. Genevaux P, Keppel F, Schwager F, Langendijk-Genevaux PS, Hartl FU, et al. (2004) In vivo analysis of the overlapping functions of DnaK and trigger factor. EMBO Rep 5: 195-200.

4. Mayer MP (1995) A New Set of Useful Cloning and Expression Vectors Derived from Pbluescript. Gene 163: 41-46.
